# High ctDNA molecule numbers relate with poor outcome in advanced ER+, HER2− postmenopausal breast cancer patients treated with everolimus and exemestane

**DOI:** 10.1002/1878-0261.12617

**Published:** 2020-02-07

**Authors:** Dinja T. Kruger, Maurice P.H.M. Jansen, Inge R.H.M. Konings, Wouter M. Dercksen, Agnes Jager, Jamal Oulad Hadj, Stefan Sleijfer, John W.M. Martens, Epie Boven

**Affiliations:** ^1^ Department of Medical Oncology Amsterdam UMC Vrije Universiteit Amsterdam/Cancer Center Amsterdam The Netherlands; ^2^ Department of Medical Oncology Erasmus MC Cancer Institute Rotterdam The Netherlands; ^3^ Department of Medical Oncology Maxima Medical Centre Eindhoven The Netherlands; ^4^ Department of Medical Oncology Gelre Ziekenhuis Apeldoorn The Netherlands

**Keywords:** biomarker, ctDNA, everolimus, metastatic breast cancer

## Abstract

We determined whether progression‐free survival (PFS) in metastatic breast cancer (MBC) patients receiving everolimus plus exemestane (EVE/EXE) varies depending on circulating tumour DNA (ctDNA) characteristics. Baseline plasma cell‐free DNA (cfDNA) from 164 postmenopausal women with ER‐positive, HER2‐negative MBC refractory to a nonsteroidal aromatase inhibitor and treated with standard EVE/EXE (Everolimus Biomarker Study, Eudract 2013‐004120‐11) was characterised for 10 relevant breast cancer genes by next‐generation sequencing with molecular barcoding. ctDNA molecule numbers, number of mutations and specific variants were related with PFS and overall survival (OS). Missense hotspot mutations in cfDNA were detected in 125 patients. The median of 54 ctDNA molecules per mL plasma distinguished patients with high and low/no ctDNA load. Patients with low/no ctDNA load (*N = *102) showed longer median PFS of 5.7 months (*P = *0.006) and OS of 124.8 months (*P = *0.008) than patients with high ctDNA load (*N = *62; 4.4 months and 107.7 months, respectively) in multivariate analyses. Patients with < 3 specific mutations (*N = *135) had longer median PFS of 5.4 months compared to those with ≥ 3 mutations (3.4 months; *P* < 0.001). In conclusion, MBC patients with low/no ctDNA load or < 3 hotspot mutations experience longer PFS while treated with EVE/EXE.

## Graphical abstract

Everolimus added to exemestane (EVE/EXE) has become standard of treatment for postmenopausal patients with estrogen receptor‐positive, human epidermal growth factor receptor type‐2‐negative metastatic breast cancer refractory to anastrozole or letrozole. Not all patients benefit from EVE/EXE and, therefore, needlessly suffer from side‐effects. This study shows that high mutant circulating tumour DNA (ctDNA) molecule numbers and ctDNA heterogeneity is associated with shorter progression‐free survival.

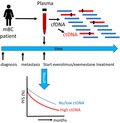

**Abbreviations**
AEs: adverse events
cfDNA: cell‐free DNA
CI: confidence interval
COSMIC: catalogue of somatic mutations in cancer
CTC‐AE: common terminology criteria for adverse events
ctDNA: circulating tumour DNA
DFI: disease‐free interval
ECOG: Eastern Cooperative Oncology Group
ER (ESR1): estrogen receptor‐α (gene)
EVE/EXE: everolimus/exemestane
HBD: healthy blood donor
HER2: human epidermal growth factor receptor 2
HR: hazard ratio
IARC: International Agency for Research on Cancer
MBC: metastatic breast cancer
METABRIC: Molecular Taxonomy of Breast Cancer International Consortium
MSK: Memorial Sloan Kettering (Cancer Center)
NGS: next‐generation sequencing
NSAI: nonsteroidal aromatase inhibitor
OS: overall survival
PFS: progression‐free survival
TCGA: The Cancer Genome Atlas

## 1 Introduction

Recently, everolimus with exemestane (EVE/EXE) has been registered for treatment for patients with estrogen receptor (ER)‐positive, human epidermal growth factor receptor type 2 (HER2)‐negative metastatic breast cancer (MBC) to prevent cancer cell survival caused by an activated PI3K pathway (Zoncu *et al.*, 2011). The BOLERO‐2 study (Baselga *et al.*, 2012) has demonstrated that patients receiving EVE/EXE had a significantly prolonged progression‐free survival (PFS) of 7.8 months compared to 4.1 months for those receiving single‐agent EXE (investigator assessment) (Yardley *et al.*, 2013). Prolonged PFS on EVE/EXE ranging from 5.6 to 9.1 months was confirmed in later studies (Jerusalem *et al.*, 2018; Moscetti *et al.*, 2016; Riccardi *et al.*, 2018; Tesch *et al.*, 2019). A proportion of patients does not benefit from the combination and will needlessly suffer from side‐effects (Rugo *et al.*, 2014). Therefore, tools are required to select patients who will likely benefit from EVE/EXE or, the reverse, withhold treatment from patients with resistant disease.

Emerging techniques enable the detection of tumour‐derived mutations in cell‐free DNA (cfDNA) from plasma of cancer patients, including breast cancer patients (De Mattos‐Arruda and Caldas, 2016). Consequently, it might be possible to link detected mutations to prognosis or therapy response. Recently, mutations in *PIK3CA* and *ESR1* have been analysed (Chandarlapaty *et al.*, 2016; Moynahan *et al.*, 2017) in cfDNA of patients in the BOLERO‐2 trial. Although *PIK3CA* mutations were detected in a substantial number of patients (43.3%), PFS was similar in EVE‐treated patients harbouring wild‐type [hazard ratio (HR) = 0.43] or mutated *PIK3CA* (HR = 0.37) (Moynahan *et al.*, 2017). Both wild‐type and mutated *ESR1* D538G patients experienced benefit from EVE/EXE (HR = 0.40 and 0.34, respectively) (Chandarlapaty *et al.*, 2016). Patients with an *ESR1* Y537S mutation had no apparent benefit from the addition of EVE, but numbers were small (Chandarlapaty *et al.*, 2016).

Unfortunately, by analysing only two or three mutations in one gene, more important mutations or multiple mutations in several genes that can predict treatment outcome might be missed. To avoid missing such valuable information, next‐generation sequencing (NGS) with molecular barcoding can be used. With this method, hotspot mutations in multiple genes can be detected simultaneously within one cfDNA analysis (Vitale *et al.*, 2019).

In the present study, cfDNA of patients that participated in the EVE Biomarker Study was analysed using NGS with molecular barcoding for the 10 most commonly affected genes in breast cancer to explore whether differences in circulating tumour DNA (ctDNA) characteristics would be associated with PFS and overall survival (OS). Characteristics included not only the number of ctDNA molecules, but also the type and frequency of mutations. The purpose of this exploratory multicentre biomarker study was to determine whether pretreatment ctDNA characterisation can be useful to select MBC patients for treatment with EVE/EXE with possible benefit.

## 2 Materials and methods

### 2.1 Study design

The EVE Biomarker Study was an exploratory, open‐label, single‐arm, multicentre study (http://ClinicalTrials.gov Identifier: NCT02109913; EudraCT number 2013‐004120‐11) to gain insight into tumour characteristics in order to predict which patients would have a high chance for a long PFS while using standard EVE/EXE. Eligible patients were ≥ 18‐year‐old postmenopausal women with ER‐positive, HER2‐negative (ER+/HER2‐) MBC and candidates for standard EVE/EXE. Their disease had to be refractory to a nonsteroidal aromatase inhibitor (NSAI) defined as a recurrence ≤ 12 months of adjuvant anastrozole or letrozole or having progressed while on or within 1 month of discontinuing NSAI treatment for metastatic disease. The NSAI did not have to be the last systemic treatment prior to enrolment. Previous treatment with mTOR inhibitors was not allowed. Patients receiving hormone replacement therapy, or those (zero)positive for HIV, hepatitis B or C or with inadequate bone marrow, liver or renal function, were excluded. All patients signed informed consent before enrolment. The study was approved by the Independent Ethics Committee of Amsterdam UMC and Institutional Review Boards at each participating site (Table S1). The study was performed in compliance with Good Clinical Practices, the Declaration of Helsinki, and carried out in keeping with applicable local law(s) and regulation(s).

Patients received EVE 10 mg and EXE 25 mg orally per day in cycles of 28 days. A starting dose of 5 mg daily for EVE was allowed to prevent stomatitis in frail patients, but in the absence of symptoms, this dose had to be increased to 10 mg in the next 2 weeks. Dose interruptions or modifications were allowed for adverse events (AEs) suspected to be related to EVE according to protocol guidelines. AEs were classified according to common terminology criteria for AEs (CTC‐AE) 4.03. AEs grade ≥ 2 were recorded in the electronic case‐record forms. Serious AEs were reported until 28 days after discontinuation of EVE unless related to progressive disease. Tumour measurements were performed with radiographic assessments to determine therapy efficacy preferably every 12 weeks.

### 2.2 Blood sample collection and cfDNA isolation

Baseline blood samples were obtained immediately before dosing of EVE/EXE. Plasma from EDTA tubes was prepared within 30 min after blood collection by centrifugation at 1500 ***g*** for 10 min at room temperature. Plasma was stored at −20 °C at the local sites until it was shipped to the central laboratory. The workflow for the isolation and NGS evaluation of cfDNA is summarised in Fig. S1. cfDNA was isolated from 2 mL plasma with a customised Maxwell^®^ (MX) RSC ccfDNA Plasma Kit (Promega, Madison, WI, USA), an automatic magnetic beads‐based method. After plasma was defrosted, a second centrifugation at 12 000 ***g*** for 10 min at room temperature was performed. In all cases, cfDNA was isolated from a starting volume of 2 mL of plasma and eluted in 60 µL of the provided elution buffer. All cfDNA isolations were performed using the manufacturer’s protocol, including a third centrifugation step at 2000 ***g*** for 10 min at room temperature to eliminate residual white blood cells. Additionally, the custom Maxwell^®^ RSC ccfDNA Plasma Kit for large plasma volume protocol was used. In brief, equal amounts of plasma and binding buffer were added together with 140 µL of magnetic beads. This mix was shaken and incubated for 45 min at room temperature and subsequently centrifuged at 2000 ***g*** for 1 min at room temperature. The pelleted mix of beads and cfDNA were transferred to the cartridge and run further on the MX instrument following standard procedures.

### 2.3 ctDNA analysis

The cfDNA of plasma from 10 healthy blood donors (HBDs) and from 171 MBC patients were analysed using the Ion Torrent™ Oncomine™ Breast cfDNA Assay in combination with the Ion Torrent S5XL Next Generation Sequencing (NGS) system, all according to protocols and consumables provided by the manufacturer (Life Technologies, Thermo Fisher Scientific, Waltham, MA, USA) (Vitale *et al.*, 2019). The cfDNA input for our HBDs ranged from 4.86 to 10.41 ng, whereas for almost all MBC patients, 10 ng cfDNA could be used to generate targeted libraries following the manufacturer’s protocol. Firstly, concentrations of each Oncomine™ cfDNA library were determined by qPCR using the Ion Library TaqMan^®^ Quantitation Kit and then diluted to a final concentration of 50 pm. Next, sample barcoded libraries were pooled together for template preparation on the Ion Chef™ (Life Technologies) Instrument using the Ion 540™ (Life Technologies) Kit – Chef and loaded onto an Ion 540™ chip. The chip was sequenced on an Ion S5™ XL Sequencer Systems, and the data were analysed using the ion torrent suite™ software 5.2.2 and torrent variant caller 5.2.1.39 (Life Technologies) and applying default software settings for low mutation frequency detection. NGS data were checked using several quality control thresholds (Fig. S1). Median read depth, median molecular coverage and mean read lengths were reported as general NGS quality measure for each cfDNA sequenced (Table S2). Samples were sequenced at a median 20 000× read depth coverage. Those cfDNA specimens with median molecule coverage below 500 molecules were excluded from further NGS analyses. The NGS data included novel and hotspot variants and were quantified by read and molecule numbers for both total and variant sequences. For this study, hotspot mutations were further analysed only when the variant itself was identified in at least three independent molecules and in 10 reads or more, and when the amplicon of the variant was sequenced for at least 300 independent molecules covered by 5000 reads or more.

The Oncomine Breast Assay sequences 26 amplicons to detect 157 hotspots and indels for a panel of 10 breast cancer relevant genes (*AKT1, EGFR, ERBB2, ERBB3, ESR1, FBXW7, KRAS, PIK3CA, SF3B1* and *TP53*; Fig. S1) as detected by ion torrent suite™ 5.2.2 and torrent variant caller 5.2.1.39 (Life Technologies). This NGS Assay applies molecular barcoding enabling the detection of mutations at allele frequencies as low as 0.1% with a recommended input of 20 ng cfDNA. Such a lower limit of detection is relevant due to the minute numbers of ctDNA molecules as demonstrated by several studies using digital PCR (Beije *et al.*, 2018; Fribbens *et al.*, 2018; Grasselli *et al.*, 2017). Routine NGS settings use allele frequencies of 1% as threshold for positivity. In our cohort of patients, this threshold would result in ctDNA detection in only 92 (56%) instead of 125 (76%) patients. Multiplex NGS with molecular barcoding also enables us to simultaneously detect multiple hotspot mutations in the 10 genes most commonly affected in breast cancer and quantify multiple different mutant molecules within one cfDNA analysis. Furthermore, it is equally sensitive as digital PCR analysis which only detects a single genetic variant in the same amount of sample.

The NGS findings for each variant were expressed as mutant ctDNA molecule numbers per mL plasma (Fig. S1), next to variant allele frequencies (VAF). ctDNA‐positive patients were defined as those with at least two (≥ 2) mutant ctDNA molecules per mL plasma, while patients with less than two (< 2) ctDNA molecules per mL plasma were called ctDNA‐negative.

### 2.4 *In silico* database analyses

The genes with mutations in cfDNA were verified in cBioPortal for their occurrence in ER+/HER2− breast carcinomas using the datasets of MK, Molecular Taxonomy of Breast Cancer International Consortium (METABRIC) and The Cancer Genome Atlas (TCGA) separately and combined (see details in Table S3). In addition, our identified cfDNA hotspot mutations were explored in catalogue of somatic mutations in cancer (COSMIC; v90) and International Agency for Research on Cancer (IARC) TP53 (v20) databases (details in Table S4). Each mutation was verified in COSMIC whether it was reported as confirmed somatic and how often it was observed in breast cancer. The *TP53* mutations were evaluated in IARC for the total somatic and germ‐line counts (Bouaoun *et al.*, 2016).

### 2.5 Statistics

Progression‐free survival was calculated as the time from the start of EVE/EXE until radiological progression of disease, clear clinical signs of progression or death by any cause. If there was no evidence of progression, but treatment was discontinued for whatever reason, patients were censored at time‐to‐treatment switch. Patients who were still on treatment at the data lock (1 March 2018) were censored at the last confirmed date of EVE/EXE exposure. OS was calculated as the time from the start of treatment until registered death; patients still alive or lost to follow‐up were censored at the last date of confirmed contact. Patients who stopped treatment with EVE/EXE within the first month were excluded from the PFS and OS analyses.

To investigate differences in ctDNA characteristics among patients with and without benefit from EXE/EVE, we divided the group into tertiles based on the duration of PFS. Each subgroup contains a third of the patients: PFS‐T1 (PFS of 2.5 months), PFS‐T2 (PFS of 5.1 months) and PFS‐T3 (PFS of 11.5 months; Table 1). To test whether the sum of specific mutations was able to distinguish survival differences in patients on treatment with EVE/EXE, an exploratory analysis was performed using cut‐off points with various numbers of mutations. A binary score of less than three (< 3) and three or more (≥ 3) specific mutations showed the clearest difference in PFS and was used in further analyses under the definition ‘number of mutations’. The median tumour load of 54 molecules per mL plasma in ctDNA‐positive patients was used as conservative threshold to distinguish patients with high ctDNA load (> 54 molecules) from those with no or low ctDNA load (0–54 molecules).

**Table 1:** Clinicopathological and cfDNA/ctDNA characteristics for the three PFS tertiles.

		Patients categorised for PFS on EVE plus EXE			
		PFS‐T1	PFS‐T2	PFS‐T3	*P*‐value^a^
Number of patients		***N* = 55** b	***N* = 55** b	***N* = 54**	
PFS (in months)	Median (range)	**2.5 (1.0**–**3.9)**	**5.1 (4.0**–**6.4)**	**11.5 (6.8**–**23.9)**	**< 0.001** ^#^
OS (in months)	Median (range)	104 (18–345)	119 (34–445)	133 (22–362)	0.13^#^
Clinicopathological characteristics					
Age	Median (range)	62 (39–90)	65 (43–90)	65 (34–75)	0.575^#^
DFI^c^	Median (range)	64 (0–274)	72 (0–304)	79 (0–301)	0.367^#^
	< 12 months, *N* (%)	6 (4)	0	2 (1)	0.136
	12–24 months, *N* (%)	11 (7)	13 (8)	12 (7)	
	> 24 months, *N* (%)	38 (23)	42 (26)	40 (24)	
(neo)Adjuvant therapy	No (neo)adjuvant therapy, *N* (%)	21 (13)	23 (14)	28 (17)	0.733
	Only chemotherapy, *N* (%)	3 (2)	4 (2)	1	
	Only endocrine therapy, *N* (%)	6 (4)	5 (3)	4 (2)	
	Both, *N* (%)	25 (15)	23 (26)	21 (13)	
PR status	Positive, *N* (%)	42 (26)	40 (24)	42 (26)	0.95
	Negative, *N* (%)	10 (6)	10 (6)	9 (5)	
	Missing, *N* (%)	3 (2)	5 (3)	3 (2)	
Metastatic sites	Bone, *N* (%)	48 (29)	50 (30)	51 (31)	0.77
	Brain, *N* (%)	2 (1)	1	2 (1)	
	Breast, *N* (%)	2 (1)	6 (4)	6 (4)	
	Liver, *N* (%)	31 (19)	25 (15)	17 (10)	
	Lung, *N* (%)	20 (12)	17 (10)	16 (10)	
	Lymph nodes, *N* (%)	19 (12)	24 (15)	16 (10)	
	Skin, *N* (%)	2 (1)	3 (2)	2 (1)	
	Other, *N* (%)	20 (12)	20 (12)	14 (9)	
Number of metastatic sites	1, *N* (%)	6 (4)	8 (5)	14 (9)	0.301
	2, *N* (%)	21 (13)	22 (13)	16 (10)	
	≥ 3, *N* (%)	28 (17)	25 (15)	24 (15)	
ECOG performance status	0, *N* (%)	19 (12)	21 (13)	24 (15)	0.636
	1, *N* (%)	33 (20)	30 (18)	29 (18)	
	2, *N* (%)	3 (2)	4 (2)	1	
Number of lines of endocrine therapy in metastatic setting^d^	0, *N* (%)	3 (2)	7 (4)	6 (4)	0.377
	1, *N* (%)	20 (12)	18 (11)	17 (10)	
	2, *N* (%)	20 (12)	16 (10)	25 (15)	
	≥ 3, *N* (%)	12 (7)	14 (9)	6 (4)	
Number of lines of chemotherapy in metastatic setting	0, *N* (%)	37 (23)	40 (24)	42 (26)	0.085
	1, *N* (%)	10 (6)	3 (2)	9 (5)	
	2, *N* (%)	3 (2)	8 (5)	3 (2)	
	≥ 3, *N* (%)	5 (3)	4 (2)	0	
cfDNA characteristics					
Amount cfDNA per mL plasma					
cfDNA (in ng)	Median (range)	12.0 (3.7–215.3)	11.3 (3.8–1595)	9.5 (4.3–331)	0.046^#^
Number of cfDNA molecules	Median (range)	1765 (0–50 808)	1122 (0–15 614)	1354 (0–160 000)	0.186^#^
ctDNA characteristics					
Amount ctDNA^e^ per mL plasma					
Variant allele frequency (VAF in %)	Median (range)	5.5 (0.0–84.3)	1.6 (0.0–65.7)	1.1 (0.0–57.0)	0.057^#^
Number of mutant ctDNA molecules	Median (range)	54 (0–12 259)	26 (0–2549)	15 (0–63 849)	0.049^#^
Patients categorised by ctDNA with:	Three or more mutations, *N* (%)	16 (10)	7 (4)	6 (4)	0.033
	> 54 ctDNA molecules (high ctDNA load), *N* (%)	27 (17)	22 (13)	13 (8)	0.024
Categorised by gene‐specific mutations^e^ in:	*PIK3CA*, *N* (%)	27 (16)	24 (15)	25 (15)	0.852
	*ESR1*, *N* (%)	27 (16)	21 (12)	17 (10)	0.172
	*TP53*, *N* (%)	12 (7)	10 (6)	15 (9)	0.490
	*SF3B1* f, *N* (%)	0	1	5 (3)	0.048
	*AKT1*, *N* (%)	1	3 (2)	1	0.533
	*ERBB2*, *N* (%)	1	1	1	1.00
	*ERBB3*, *N* (%)	1	1	1	1.00
	*KRAS*, *N* (%)	0	1	1	1.00
	*EGFR*, *N* (%)	0	0	0	1.00
	*FBXW7*, *N* (%)	0	0	0	1.00

a *P*‐values for the comparison of the three PFS groups for EVE and EXE are based on a chi‐square test for *r* × *c* contingency tables as calculated with http://www.physics.csbsju.edu/cgi-bin/stats/contingency; *P*‐values with ^#^ are based on a test for trend calculated by stata, StataCorp LLC (College Station, TX, USA).

b Both PFS‐T1 and PFS‐T2 had each three patients with no event for PFS due to toxicity or no clinical benefit after one cycle EVE/EXE therapy.

c DFI is defined as the time from diagnosis of primary breast cancer to first relapse in months. All patients but one had stage IV disease at presentation.

d Different aromatase inhibitors were counted as separate lines.

e Cases were called ctDNA‐positive when at least two mutant ctDNA molecules per mL plasma were detected for any gene or for a specified gene.

f Two cases had only three *SF3B1*‐mutant molecules per mL plasma; the other four cases had 11, 15, 20 and 52 mutant molecules per mL plasma.

Significance was defined at < 0.05.

Tests for trends, Kruskal–Wallis and chi‐square were performed for nonparametric analyses of continuous or categorical variables and used as indicated in Tables. To analyse which ctDNA characteristics related with PFS or OS, multivariate step‐down analyses were performed for ctDNA characteristics with at least 10% patient cases per characteristic. Uni‐ and multivariate Cox regression analyses were used to calculate HR, 95% confidence intervals (95% CI) and *P*‐values. Clinicopathological factors included in the multivariate analyses were age, disease‐free interval (DFI), visceral metastasis, (neo)adjuvant therapy, number of treatment lines for metastatic disease, progesterone receptor (PR) and Eastern Cooperative Oncology Group (ECOG) screening visit status. *P*‐values were two‐sided and significance was defined at < 0.05. Survival time analyses were visualised by Kaplan–Meier curves; log‐rank test was applied to test for differences between survival curves. The study complied with reporting recommendations for tumour marker prognostic studies (REMARK) criteria (McShane *et al.*, 2005). Statistical analyses were generated with spss 22.0 (IBM SPSS, Armonk, NY,USA) and STATA 14 (StataCorp LLC, College Station, TX, USA).

## 3 Results

Details on inclusion and exclusion criteria, EVE/EXE dosing, AEs management, plasma preparation, cfDNA isolation, Ion Torrent^TM^ NGS are described in the Appendix S1.

### 3.1 Patients and adverse events

A total of 178 patients signed informed consent between March 2014 and February 2017 in 28 participating hospitals in the Netherlands (Table S1). Two patients were excluded who did not meet the inclusion and exclusion criteria, and one patient was excluded who never started treatment (Fig. S2; Table S5). Median PFS was 5.3 months (95% CI: 4.77–5.87) ranging from 0.46 to > 36.8 months.

At the data lock on 1 March 2018, five patients were still on treatment. Reasons for discontinuation other than progressive disease were as follows: toxicity (*N = *13), physician’s decision (*N = *2) and one at request of the patient. Median age of the study participants was 63 years. Most patients had metastases involving two or more sites (82%); 26 patients (15%) had bone only disease. At the time of the primary diagnosis, 128 (73%) tumours were PR positive. Thirty‐six patients (21%) presented with advanced disease as first breast cancer diagnosis. Most patients received prior systemic treatment for their metastatic disease; for 17 patients (10%), EVE/EXE was given as first‐line therapy in the metastatic setting.

In total, 383 AEs grade ≥ 2 occurred, which were possibly, probably or definitely related to EVE. The most common AEs grade ≥ 2, either possibly, probably or definitely related to EVE are listed in Table S6. There were three on treatment deaths not related to EVE/EXE. One patient died from pneumonitis related to EVE in the follow‐up period of 28 days.

Next‐generation sequencing data could be generated for 164 out of 175 patients (Figs S1 and S2; Table S5). Reasons for exclusion were as follows: no baseline plasma available (*N* = 5), insufficient NGS quality (*N* = 2) and discontinuation of treatment within cycle 1 due to toxicity (*N* = 4). Clinicopathological characteristics of the 164 patients are shown in Table S5.

### 3.2 Occurrence of mutations

**Figure 1:**
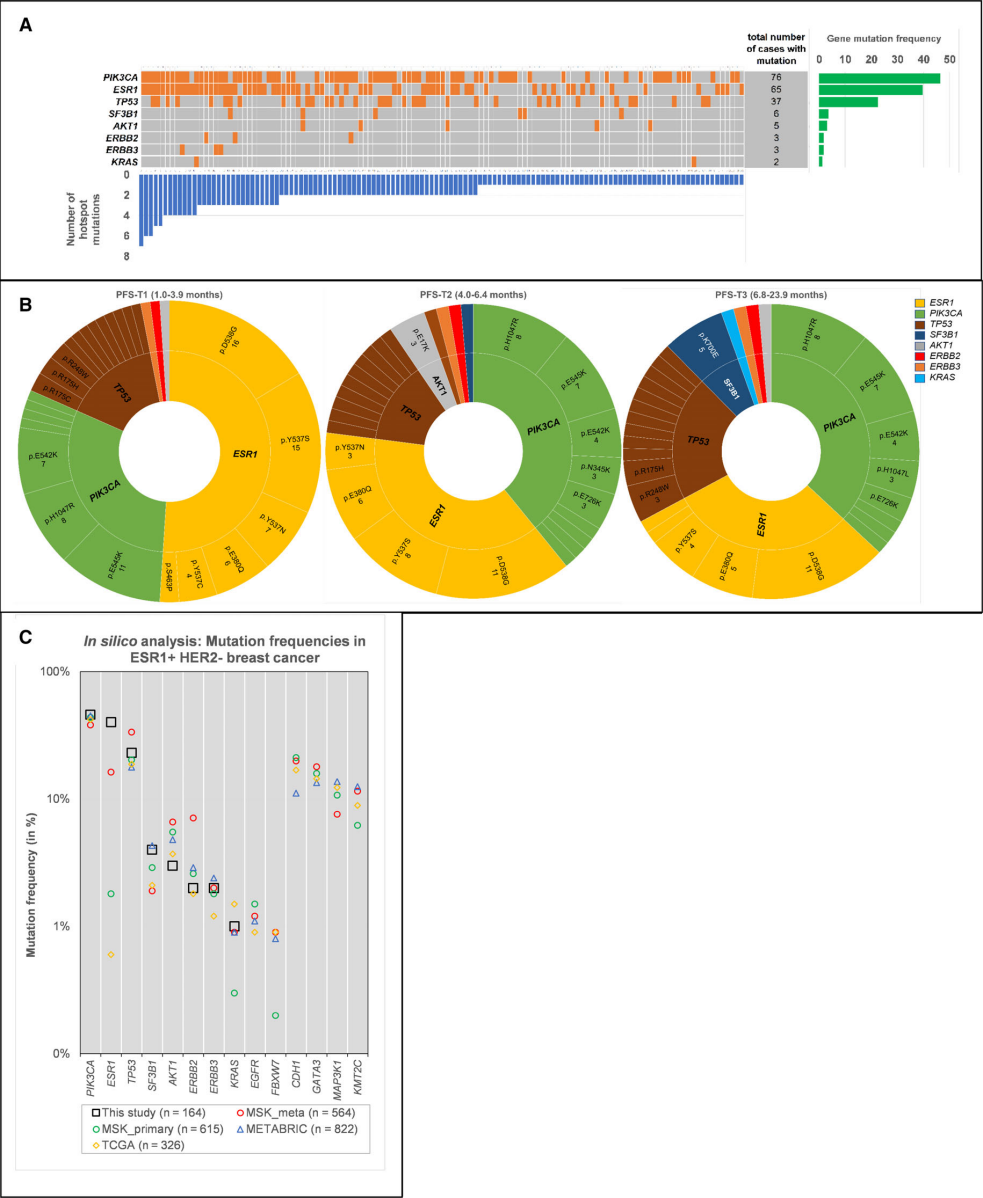
Mutational landscape of this study. (A) Landscape plot summarising 125 patients with gene mutations (orange boxes) detected in cfDNA by the Oncomine NGS panel. Number of hotspot mutations is illustrated by the blue vertical bars and the number of patients with a gene mutation by the green bars. (B) Sunburst plots for gene hotspot mutations identified in patients grouped per PFS tertile. Genes and hotspot mutations are ordered clockwise from high to low incidence. *ESR1* hotspot mutations, especially p.Y537S, are most frequent in patients with poor response to EVE/EXE (PFS‐T1). The *SF3B1* mutations are mainly observed in patients with benefit from EVE/EXE (PFS‐T3). (C) *In silico* analyses of ER+/HER2− breast carcinomas using the cBioPortal datasets MSK, METABRIC, TCGA. The Oncomine cfDNA panel genes and the most frequently mutated genes of each dataset are shown. Only the *ESR1* mutation frequency in our study is considerably higher than that within the other datasets.

Most patients had mutations in *PIK3CA* [76/164 (46%)], *ESR1* [65/164 (40%)], and *TP53* [37/164 (23%)] (Fig. 1A, Table S3). Mutations were rare in *SF3B1* [6/164 (4%)], *AKT1* [5/164 (3%)], *ERBB2* [3/164 (2%)], *ERBB3* [3/164 (2%)], *KRAS* [2/164 (1%)] and were not detected in *EGFR* and *FBXW7*. The most frequently detected variants (Fig. 1B, Table S4) resulting in oncogenic amino acid changes in *ESR1* were p.D538G (*N = *38), p.Y537S (*N = *27) and p.E380Q (*N = *17). For *PIK3CA,* these were p.E545K (*N = *25), p.H1047R (*N = *24) and p.E542K (*N = *15).

The TCGA, METABRIC and Memorial Sloan Kettering (MSK) datasets were explored *via* cBioPortal for *in silico* analyses of the mutational landscape of primary or metastatic biopsies of ER+/HER2‐ breast carcinomas. Mutational frequencies of all 10 genes used in the Oncomine cfDNA panel and additional genes representing the most frequently mentioned genes in each dataset are shown in Fig. 1C and Table S5. Of the analysed genes, the mutation frequency of only *ESR1* was considerably higher in our study than in the consulted datasets. The 26 *TP53* mutations detected in our study were verified in the IARC TP53 (v20) and COSMIC (v90) database for germ‐line reports (Table S5). Of these, 22 *TP53* mutations were mentioned as germ‐line and only four mutations (p.C238F, p.H179L, p.L194R and p.R249M) were not. The 22 mutations with germ‐line counts in IARC were reported as confirmed somatic mutations, and 17 of these have frequently been observed in breast cancer by COSMIC. Thus far, germ‐line mutations for *PIK3CA* have not yet been reported.

### 3.3 Relationship between ctDNA characteristics and PFS

A total of 125 of 164 patients (76%) were considered ctDNA‐positive, because they had at least two mutant ctDNA molecules per mL plasma with one (*N = *55) or more (*N = *70) missense hotspot mutations (Fig. S3). The median tumour load in ctDNA‐positive patients was 54 molecules per mL plasma (range 2–2549; Table S7). This median was used as conservative threshold to distinguish patients with high ctDNA load of > 54 molecules (*N = *62; 38%) and those with no or low ctDNA load (*N = *102, 62%). Most patients discontinued treatment due to progression of disease, although some discontinued EVE earlier than EXE. Total duration of EVE exposure and PFS correlated strongly (*R* = 0.95, data not shown), because of which time of treatment will not change our findings. The total dose of EVE correlated strongly with PFS (*R* = 0.89) and Cox regression demonstrated that this depended on ctDNA load (HR = 0.99, 95% CI: 0.99–1.00, *P* < 0.001; data not shown).

**Figure 2:**
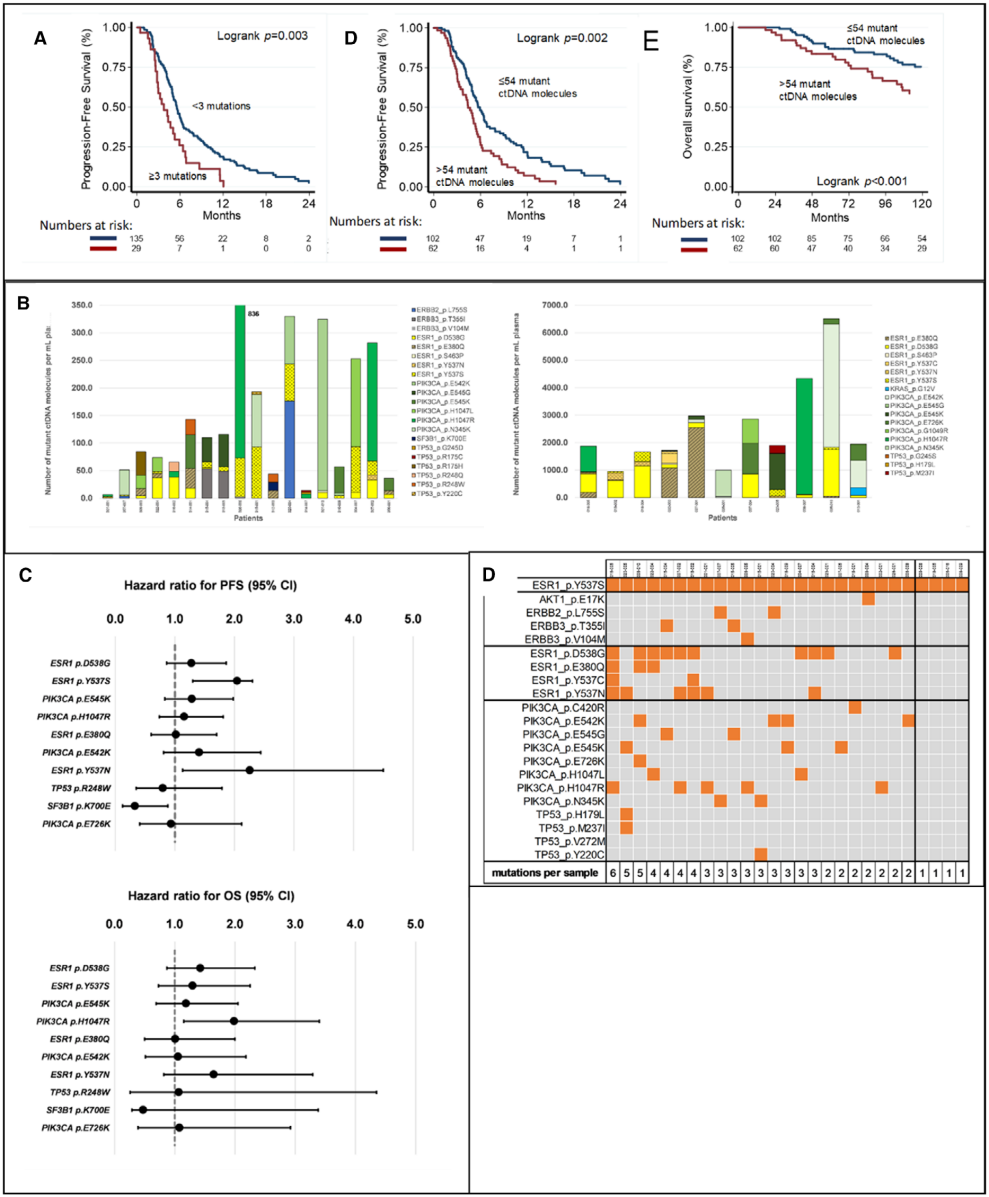
ctDNA characteristics and survival. (A) The ctDNA load and number of mutations and their relation with PFS on EVE/EXE and with OS. (B) Samples (*n* = 29) with ctDNA containing ≥ 3 mutations showing heterogeneity in mutant ctDNA molecules per patient. It represents the sum of mutant ctDNA molecules per mL plasma for all gene mutations found in 29 patients with ≥ 3 hotspot mutations in their ctDNA. The figure shows the patients who have < 1000 (left, *N* = 18) or more than 1000 (right, *N* = 11) mutant ctDNA molecules per mL plasma. Some patients exhibit clearly large differences in the number of mutant ctDNA molecules among mutations. (C) Top 10 most frequent gene hotspot mutations observed in this study and relationship with PFS and OS. (D) Patients with *ESR1* p.Y537S mutations (*n* = 27) have no other mutation (*n* = 4), additional mutations in *ESR1* (*n* = 12), or mutations in other genes (*n* = 11).

Patient groups were discriminated having ≥ 3 (*N* = 29) or < 3 (*N* = 135) specific mutations. Within single patients with different mutations detected, the number of ctDNA molecules among specific mutations could vary considerably (Fig. 2B). Multivariate step‐down analysis (Table S8) revealed that patients with ctDNA containing ≥ 3 mutations (3.4 months, *P = *0.033) or with high ctDNA load (4.4 months, *P = *0.024) had significantly shorter median PFS than patients with fewer mutations or with no/low ctDNA load (5.4 months and 5.7 months, respectively; Fig. 2A). This was confirmed in uni‐ as well as multivariate analyses (Table 2, Table S9) and illustrated by Kaplan–Meier curves (Fig. 2A). Of interest, the number of mutations and ctDNA load combined correlated more strongly with PFS than each separate factor in both uni‐ and multivariate analysis as shown in Table 2.

**Table 2:** Uni‐ and multivariate analyses of clinicopathological factors and ctDNA load.

	*N*	PFS							OS						
			Univariate analysis			Multivariate analysis				Univariate analysis			Multivariate analysis		
		Events	HR	95% CI	*P*‐value	HR	95% CI	*P*‐value	Events	HR	95% CI	*P*‐value	HR	95% CI	*P*‐value
Age (in years)															
≤ 55	34	31	1.00			1.00			16	1.00			1.00		
56–70	91	84	0.94	0.62–1.42	0.775	0.91	0.60–1.39	0.671	43	0.65	0.36–1.17	0.146	0.99	0.53–1.87	0.996
> 70	39	36	1.64	1.01–2.68	0.047	1.65	0.99–2.76	0.056	25	0.82	0.43–1.57	0.549	1.47	0.73–2.98	0.280
DFI (in months)															
≤ 12	44	40	1.00			1.00			24	1.00			1.00		
> 12	120	111	0.98	0.68–1.41	0.903	0.62	0.40–0.97	0.038	60	0.10	0.06–0.18	< 0.001	0.06	0.03–0.12	< 0.001
Visceral metastasis															
No	48	41	1.00			1.00			21	1.00			1.00		
Yes	116	110	1.45	1.01–2.08	0.043	1.49	1.02–2.17	0.040	63	0.91	0.55–1.50	0.717	1.38	0.81–2.36	0.234
(Neo)Adjuvant therapy															
No	72	63	1.00			1.00			40	1.00			1.00		
Yes	92	88	1.31	0.95–1.82	0.102	1.70	1.14–2.54	0.010	44	0.78	0.50–1.19	0.246	1.59	0.95–2.64	0.076
Number of lines of therapy for metastatic disease															
≤ 2	111	101	1.00			1.00			50	1.00			1.00		
> 2	53	50	1.31	0.93–1.84	0.126	1.42	0.99–2.04	0.057	34	1.13	0.73–1.76	0.576	0.77	0.48–1.25	0.296
PR status primary															
Negative/unknown^a^	40	37	1.00			1.00			19	1.00			1.00		
Positive	124	114	0.84	0.57–1.21	0.344	0.85	0.58–1.24	0.402	65	1.14	0.68–1.92	0.621	1.45	0.84–2.48	0.182
ECOG screening visit status															
ECOG = 0	64	59	1.00			1.00			28	1.00			1.00		
ECOG^b^ = 1 or 2	100	92	1.25	0.90–1.75	0.178	1.10	0.77–1.55	0.607	56	1.19	0.75–1.87	0.459	1.52	0.94–2.46	0.085
Number of mutant ctDNA molecules per mL plasma															
≤ 54 molecules (no/low ctDNA load)	102	91	1.00			1.00			41	1.00			1.00		
> 54 molecules (high ctDNA load)	62	60	1.66	1.19–2.31	0.003	1.64	1.16–2.33	0.006	43	2.20	1.42–3.39	< 0.001	1.83	1.17–2.87	0.008
Number of hotspot mutations															
< 3 mutations	135	123	1.00			1.00			64	1.00					
≥ 3 mutations	29	28	1.86	1.22–2.83	0.004	2.20	1.43–3.38	< 0.001	20	1.50	0.91–2.49	0.112	1.62	0.97–2.70	0.067
Combined ctDNA load and number of hotspot mutations															
Both low	91	80	1.00			1.00			34	1.00			1.00		
High/low or low/high	55	54	1.53	1.08–2.18	0.017	1.58	1.10–2.29	0.014	37	2.54	1.58–4.09	< 0.001	2.37	1.44–3.91	0.001
Both high	18	17	2.79	1.63–4.78	< 0.001	2.78	1.61–4.79	< 0.001	13	2.10	1.10–3.99	0.024	1.82	0.95–3.52	0.069

a PR status is unknown for 11 patients.

b 92 patients with ECOG = 1, 8 patients with ECOG = 2.

### 3.4 ctDNA characteristics in three PFS tertiles

We compared ctDNA characteristics among three subsets of patients grouped in tertiles based on PFS period. These three subsets were similar for clinicopathological factors (Table 1, Fig. 1B). Patients in PFS‐T3 had preferentially less ctDNA molecules (median 15) than patients in PFS‐T2 (median 26) and PFS‐T1 (median 54). *SF3B1* mutations were preferentially observed in patients in PFS‐T3 (Table 1, Fig. 1B). Patients with shorter PFS from EVE/EXE had relatively more ctDNA containing *ESR1* mutations compared to those with benefit (Fig. 1B). Specifically, *ESR1* variants p.Y537S (*P = *0.023), p.Y537N (*P = *0.084), and p.Y537C (*P = *0.088) were preferentially observed in patients in PFS‐T1 (Fig. 1B, Table S4). Univariate Cox regression analyses confirmed these findings (Fig. 2C, Table S10). Since *ESR1* p.Y537S was one of the most frequently observed mutations (*n* = 27) and especially in patients with short PFS, it was investigated in more detail (Fig. 2D). In four patients, *ESR1* p.Y537S was the only hotspot mutation identified. Twelve patients had additional *ESR1* mutations, whereas the remaining eleven patients had one or more mutations in other genes.

### 3.5 Multivariate analyses of ctDNA load with clinicopathological factors

Uni‐ and multivariate Cox regression analyses of clinicopathological factors, ctDNA load and number of hotspot mutations for PFS and OS are presented in Table 2. Clinicopathological factors associated with a worse PFS in the univariate analyses were age > 70 years (*P = *0.047) and visceral metastases (*P = *0.043). In the multivariate analyses, presence of visceral metastases and (neo)adjuvant therapy turned out to be significantly associated with a worse PFS, while longer DFI was associated with a better PFS. The only clinicopathological factor associated with longer OS was DFI in both univariate and multivariate analyses (both *P* < 0.001). Number of mutations and ctDNA load were both independently related with a worse PFS in uni‐ as well as multivariate analyses (Table 2). With regard to OS, ctDNA load was significantly related with a worse survival (uni *P < *0.001, multi *P = *0.008).

### 3.6 Relationship between ctDNA characteristics and overall survival

Shorter OS was observed in patients with high ctDNA load compared to low ctDNA load patients (HR = 2.20, *P* < 0.001; Fig. 2A). Shorter OS was also found in patients with a *PIK3CA* mutation (HR = 1.80, *P = *0.011) and especially in those with a p.H1047R mutation (HR = 1.98, *P = *0.013; Fig. 2C, Table S10). Step‐down analyses revealed that only high ctDNA load remained associated with a shorter OS (Table S8) as illustrated by the Kaplan–Meier survival curve (Fig. 1E; *P < *0.001). Uni‐ and multivariate Cox regression analyses for OS confirmed that only high ctDNA load was significantly associated with a worse survival (uni *P < *0.001, multi *P = *0.008, Table 2). OS in patients with ≤ 54 ctDNA molecules was 124.8 months, while that in patients with > 54 ctDNA molecules was 107.7 months. As shown in Table 2, combining ctDNA load with number of mutations resulted in a stronger association with OS in both uni‐ and multivariate analysis.

## 4 Discussion

In daily clinical practice, MBC patients being candidates for standard EVE/EXE will present a variety of prognostic factors. Characterisation of ctDNA at baseline might be a less invasive way to eventually help selecting patients who will likely experience benefit from EVE/EXE. In the present study, we demonstrated that patients with low or no ctDNA load had longer PFS than those with > 54 ctDNA molecules·mL^−1^. Longer PFS was also observed in patients with plasma containing < 3 specific mutations. Especially patients with no or low ctDNA load and < 3 mutations had longer PFS.

Up to now, many NGS studies report allele frequencies as parameter to quantify ctDNA. The allele frequency is the ratio of the number of mutant alleles divided by the number of wild‐type alleles with the mutant alleles being derived from tumour cells only, but wild‐type alleles originate from both tumour as well as normal cells. Importantly, our previous analyses showed that allele frequencies are substantially affected by preanalytical conditions, in particular by inducing lysis of leucocytes causing higher numbers of wild‐type alleles, while numbers of mutant ctDNA variants remain stable (van Dessel *et al.*, 2017). As a consequence, we decided to report only the number of mutant molecules per mL plasma.

In our study, we found that the number of ctDNA molecules varied for different mutations detected within a single patient. These interpatient ctDNA number variances are suggestive for the existence of major and minor tumour cell subclones. In the course of the disease, ctDNA may not only increase due to higher tumour burden, but also from minor subclones expanding from heterogeneous tumours that consequently may cause therapy resistance. This is underlined by the finding that *ESR1* mutations in ctDNA are generally found in MBC patients after exposure to aromatase inhibitors and that these mutations predict aromatase inhibitor resistance (Jeselsohn *et al.*, 2017). In addition, ctDNA profiling in lung cancer patients revealed mutational heterogeneity between pre‐ and post‐treatment samples, while the type of mutations depended on the therapy given (Chabon *et al.*, 2016).

Possible explanations for a worse PFS in the presence of high ctDNA levels might thus be that this reflects higher tumour burden, while the presence of different mutations might point towards the development of treatment‐resistant clones. We are the first to report this finding in a cohort of ER+/HER2‐ MBC patients treated with EVE/EXE. The relationship between ctDNA and prognosis has more broadly been studied. In a recent meta‐analysis, Lee *et al. *(2018) have reported that the ctDNA mutation rate measured in plasma of breast cancer patients predicts disease recurrence and unfavourable survival outcomes. In a small number of 26 MBC patients, Dawson *et al. *(2013) have shown that increasing levels of ctDNA were associated with a worse prognosis as well as with progressive disease. In our cohort of patients, high ctDNA levels were also prognostic for poor OS. Whether candidates for EVE/EXE with poor ctDNA characteristics have more benefit from alternative treatment, such as chemotherapy, should be subject of further studies. Furthermore, it would be interesting to analyse ctDNA levels at sequential time‐points during treatment and assess whether changes are associated with clinical outcome on EVE/EXE.

Contrary to mutational load and total number of mutations, we found no effect of the single mutations on PFS except for *SF3B1* and *ESR1* p.Y537S. Patients with a mutation in *SF3B1*, a gene encoding an mRNA splicing factor, were more frequently found in the longer PFS subgroup PFS‐T3 compared to those without the mutation. This is in accordance with previous work reporting the *SF3B1* mutation is predominantly found in the luminal A subtype of breast cancer, a subgroup known to have a relatively better outcome than other breast cancer patients (Cancer Genome Atlas Network, 2012; Ellis *et al.*, 2012). Patients with an *ESR1* p.Y537S mutation were mainly found in PFS‐T1 with shorter PFS. This is in line with a subgroup analysis from patients who participated in the BOLERO‐2. In that analysis, the *ESR1* p.Y537S mutation in ctDNA was significantly associated with a shorter OS (Chandarlapaty *et al.*, 2016). However, these associations were only found in the PFS tertile subgroup analyses, while high numbers of ctDNA molecules and multiple specific mutations were independently associated with PFS in the multivariate analyses. We, therefore, believe that the latter factors are better associated with PFS than the single mutations.

This study has some limitations. First, median PFS of 5.3 months in our study was shorter than the 7.8 months presented in the BOLERO‐2 study (Yardley *et al.*, 2013) and the 8 months in the study of Moscetti *et al. *( 2016). Less stringent inclusion and exclusion criteria are required in a general population of patients being candidates for EVE/EXE. In the 4EVER trial allowing broader inclusion criteria, a PFS similar to ours of 5.6 months was reached (Tesch *et al.*, 2019). Second, there was no control group receiving EXE plus placebo. The inclusion of such a control group was considered unethical since the BOLERO‐2 study had demonstrated that PFS on EVE/EXE was superior to EXE monotherapy in all subgroups. Therefore, we were not able to determine whether ctDNA characterisation is useful to predict true benefit from EVE/EXE. It would be interesting if our results could be reproduced in the BOLERO‐2 study population to distinguish the prognostic and predictive value of ctDNA biomarkers. Third, neither the 10‐gene panel nor a similar tool has been used before to analyse the effect of multiple mutations or mutational load on PFS of patients using standard EVE/EXE. Whether other than these 10 genes add to the mutational load is not yet known. The genes selected for our assay, however, are frequently mutated in breast cancer and the selected single nucleotide variants and short indels cover > 150 hotspot mutations. Last, our cohort of patients might have a different genomic make‐up in metastases than that in primary breast cancer, as shown by the *in silico* analyses. In that respect, Angus *et al. *(2019) have recently reported on the genomic landscape of MBC and showed more frequent mutations in *ESR1, TP53, NF1, AKT1, KMT2C and PTEN* in ER+/HER2‐ metastatic lesions than in primary breast carcinomas. Our targeted assay evaluated three of these genes (*ESR1, TP53 and AKT1*). Previous groups have assessed mutations in only one or two genes, but did not report a clear effect on PFS (Chandarlapaty *et al.*, 2016; Moynahan *et al.*, 2017). Our study shows that ctDNA load and number of mutations separately and combined clearly associate with PFS from standard EVE/EXE in MBC patients.

## 5 Conclusions

Our ctDNA analyses using targeted NGS combined with molecular barcoding of cfDNA showed that MBC patients treated with EVE/EXE and with no or low ctDNA load in pretreatment plasma had a prolonged PFS. Patients with shorter survival while being treated with standard EVE/EXE were characterised by high numbers of ctDNA molecules and ≥ 3 specific mutations. The *ESR1* p.Y537S mutation was associated with a shorter survival, while mutations in *PIK3CA* were not related with outcome. Whether ctDNA characteristics are useful for screening patients likely not to be treated with EVE/EXE, thereby avoiding unnecessary toxicity and financial costs, should be confirmed in an independent study.

## Conflict of interest

The authors declare no conflict of interest.

## Author contributions

DK, MJ, SS, JM and EB designed the study and drafted the manuscript. DK, IK, MD, AJ, JOH, SS and EB were responsible for acquiring patients and collecting clinical data. MJ, DK and JM were involved in the methodology development, database construction and (statistical) data analyses. DK and MJ wrote the manuscript; IK, MD, AJ, JOH, SS, JM and EB reviewed and revised the manuscript. The study was supervised by SS and EB.

## Consent to publish

The authors are fully responsible for the contents of this manuscript, and the views and opinions described in the publication reflect solely those of the authors.

## Supporting information


**Fig. S1.** Biomarker workflow: Plasma cfDNA isolation and ctDNA characterization by NGS and molecular barcoding.

**Fig. S2.** Study design: Setting and participants of the EVE plus EXE Biomarker study.

**Fig. S3.** ctDNA characteristics: Number of mutations and ctDNA load.

**Fig. S4.** Kaplan‐Meier survival curves evaluation for ctDNA and its relationship with PFS on EVE/EXE.

**Table S1.** List of participating hospitals.

**Table S2.** Summary and details of NGS results.

**Table S3.** *In silico* database evaluation of Oncomine cfDNA panel genes and most frequently mutated genes of each dataset.

**Table S4.** List of identified gene hotspot mutations, their occurrence in EVE/EXE response subsets, and their COSMIC and IARC information.

**Table S5.** Clinical and cfDNA characteristics of total study population and patients with NGS data.

**Table S6.** Summary of AEs possibly, probably or definitely related to EVE.

**Table S7.** Summary of cfDNA and ctDNA characteristics.

**Table S8.** Multivariate stepdown analysis.

**Table S9.** Uni‐ & multivariate analysis of ctDNA characteristics for progression‐free and OS.

**Table S10.** Cox regression analyses gene hotspot mutations for progression‐free and OS.

**Appendix S1.** Results.

## Data Availability

All data are available in supplementary files.
