# Supplementary figures and images for: High ctDNA molecule numbers relate with poor outcome in advanced ER+, HER2− postmenopausal breast cancer patients treated with everolimus and exemestane
Source: Mol Oncol. 2020 Feb 7;14(3):490–503. doi: 10.1002/1878-0261.12617 (PMC7053245; doi:10.1002/1878-0261.12617)

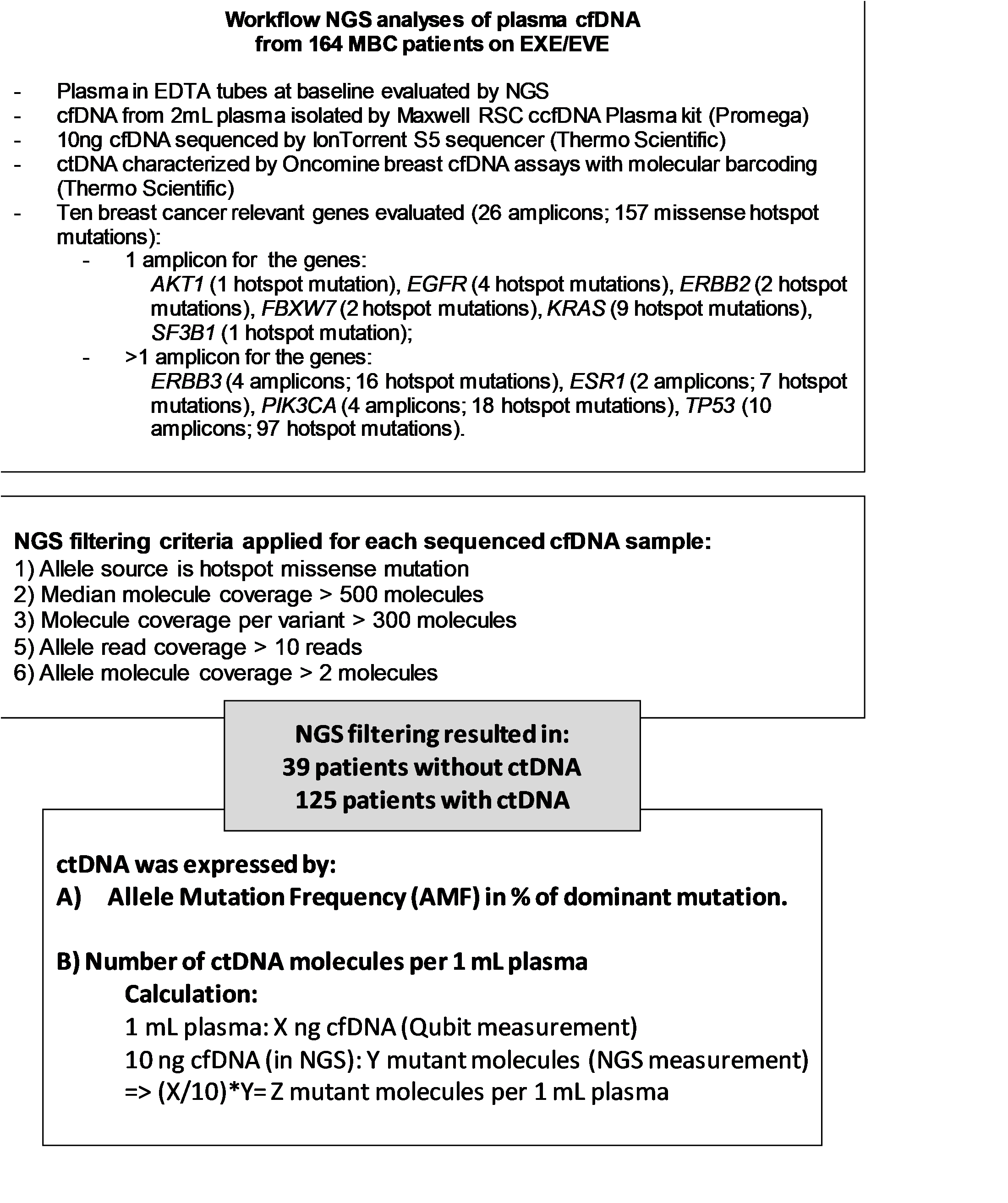

Supplement: Supplementary file 1 — Fig. S1. Biomarker workflow: Plasma cfDNA isolation and ctDNA characterization by NGS and molecular barcoding. [file MOL2-14-490-s001.png]

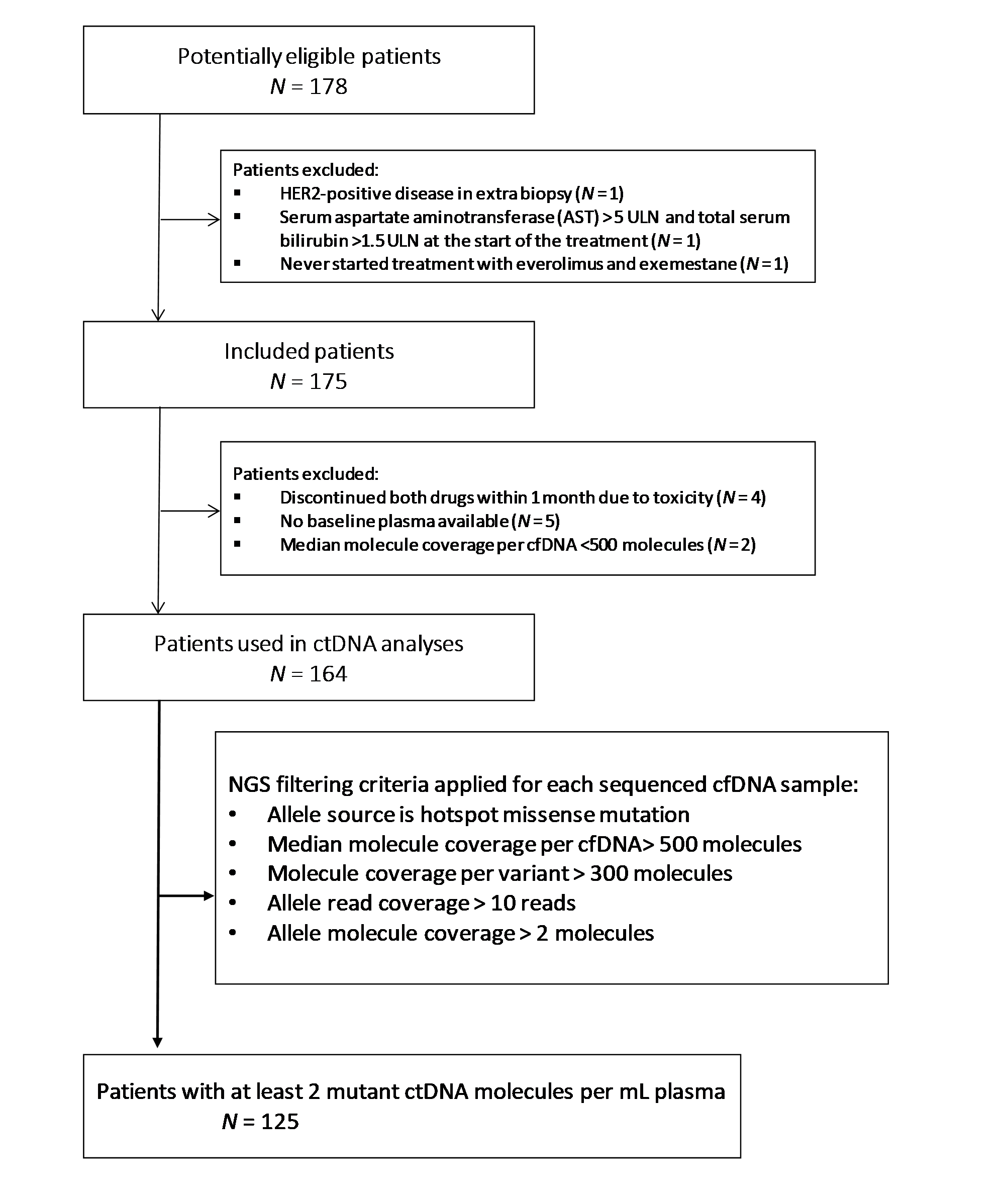

Supplement: Supplementary file 2 — Fig. S2. Study design: Setting and participants of the EVE plus EXE Biomarker study. [file MOL2-14-490-s002.png]

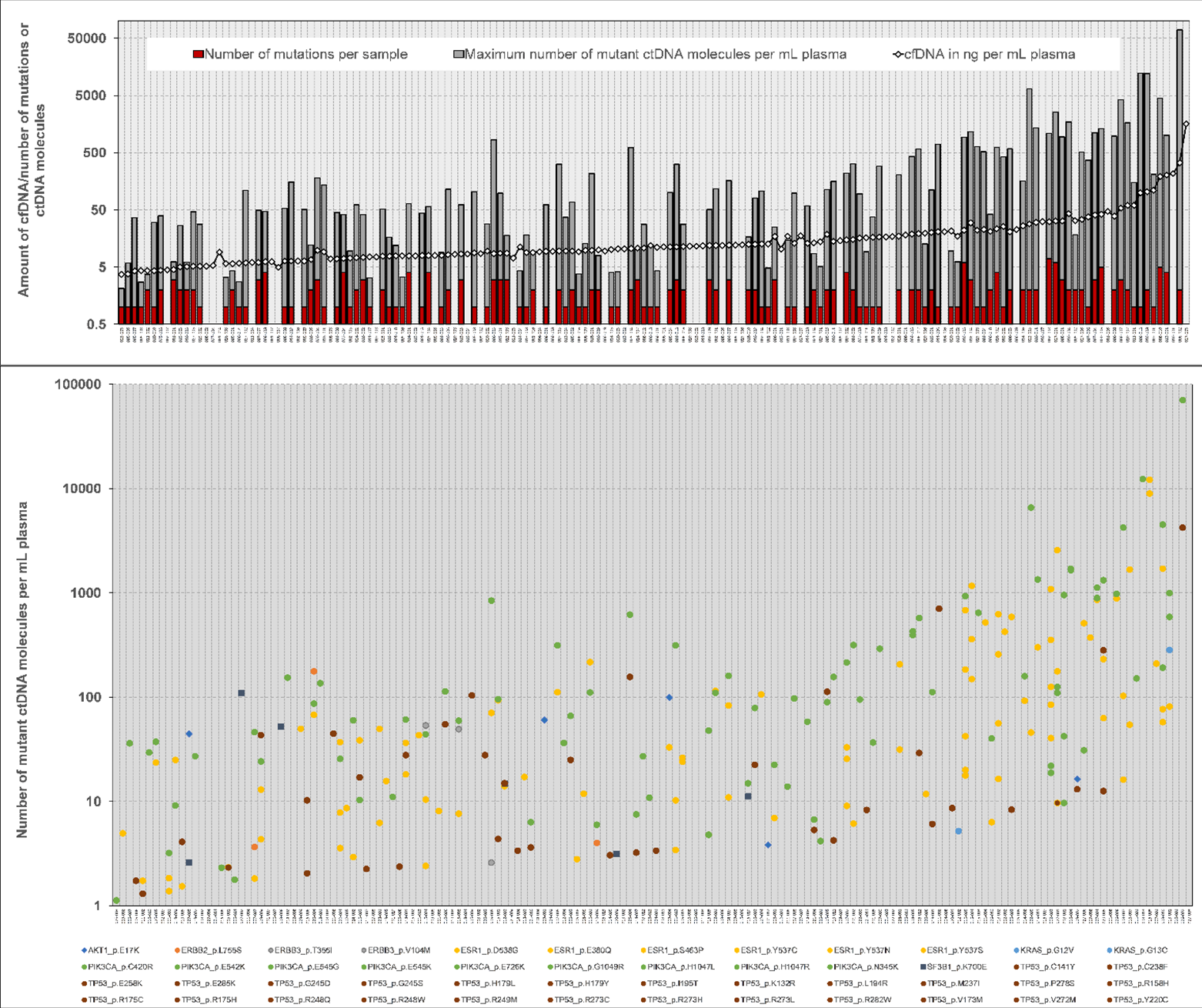

Supplement: Supplementary file 3 — Fig. S3. ctDNA characteristics: Number of mutations and ctDNA load. [file MOL2-14-490-s003.png]

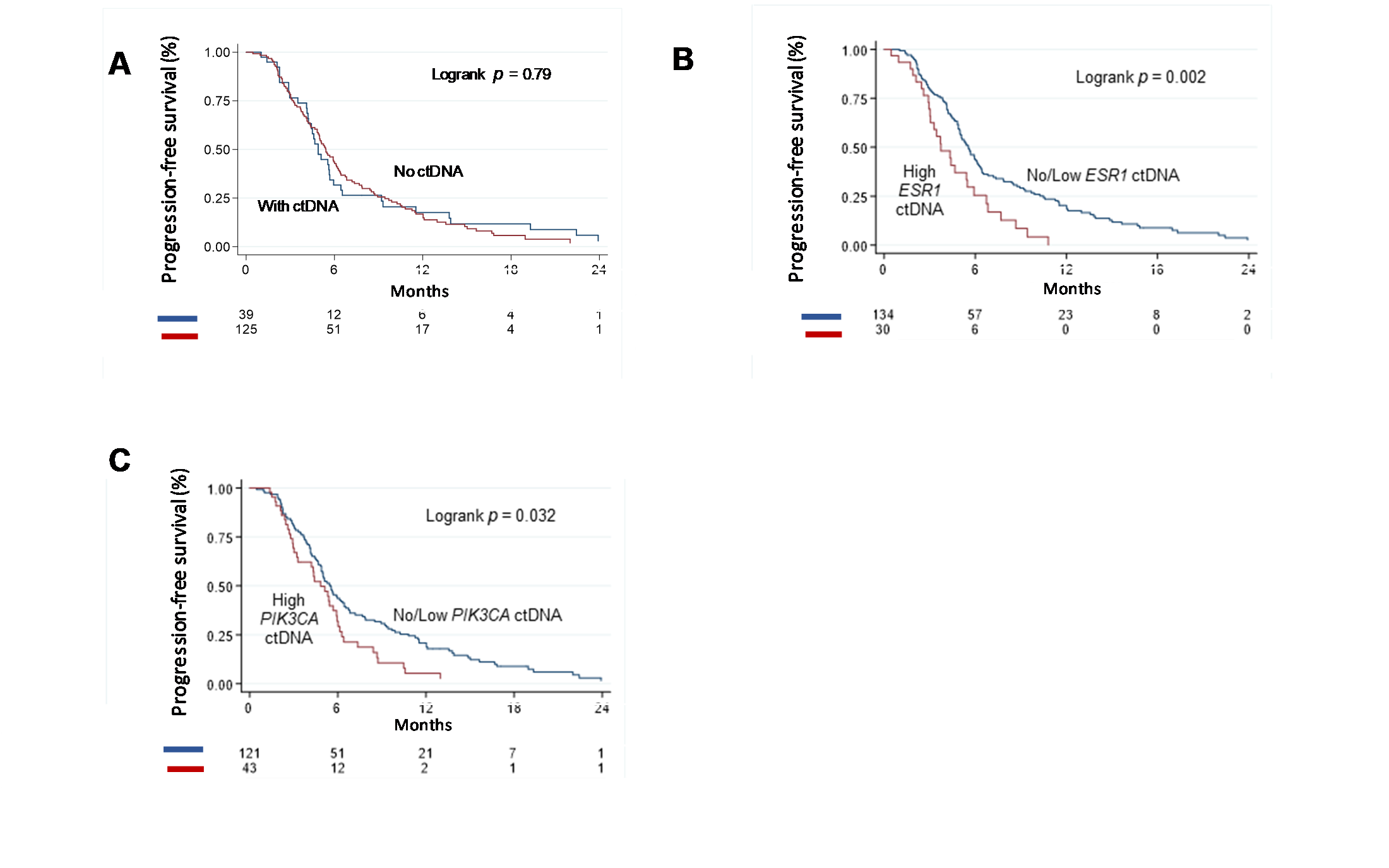

Supplement: Supplementary file 4 — Fig. S4. Kaplan‐Meier survival curves evaluation for ctDNA and its relationship with PFS on EVE/EXE. [file MOL2-14-490-s004.png]

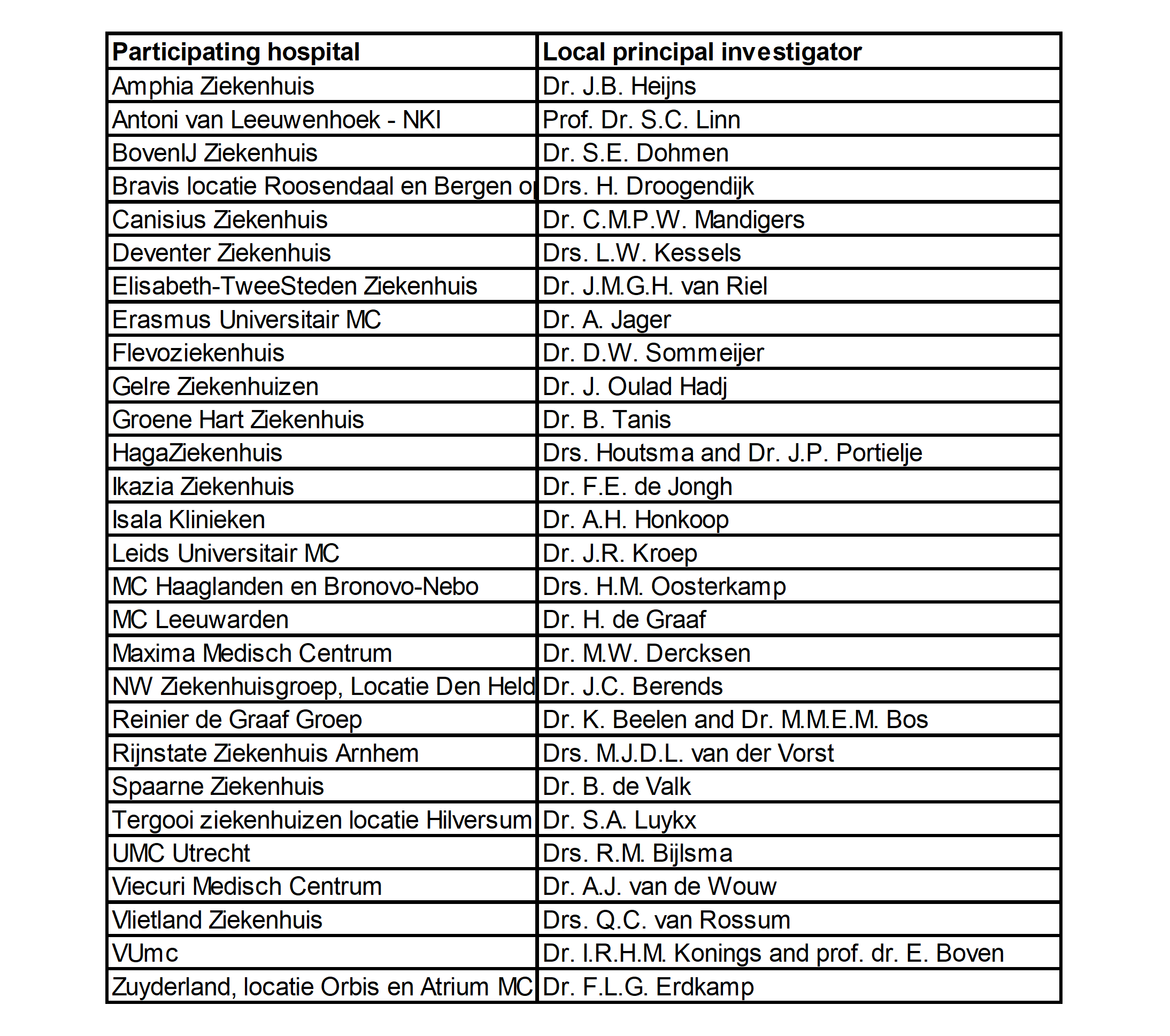

Supplement: Supplementary file 5 — Table S1. List of participating hospitals. [file MOL2-14-490-s005.png]

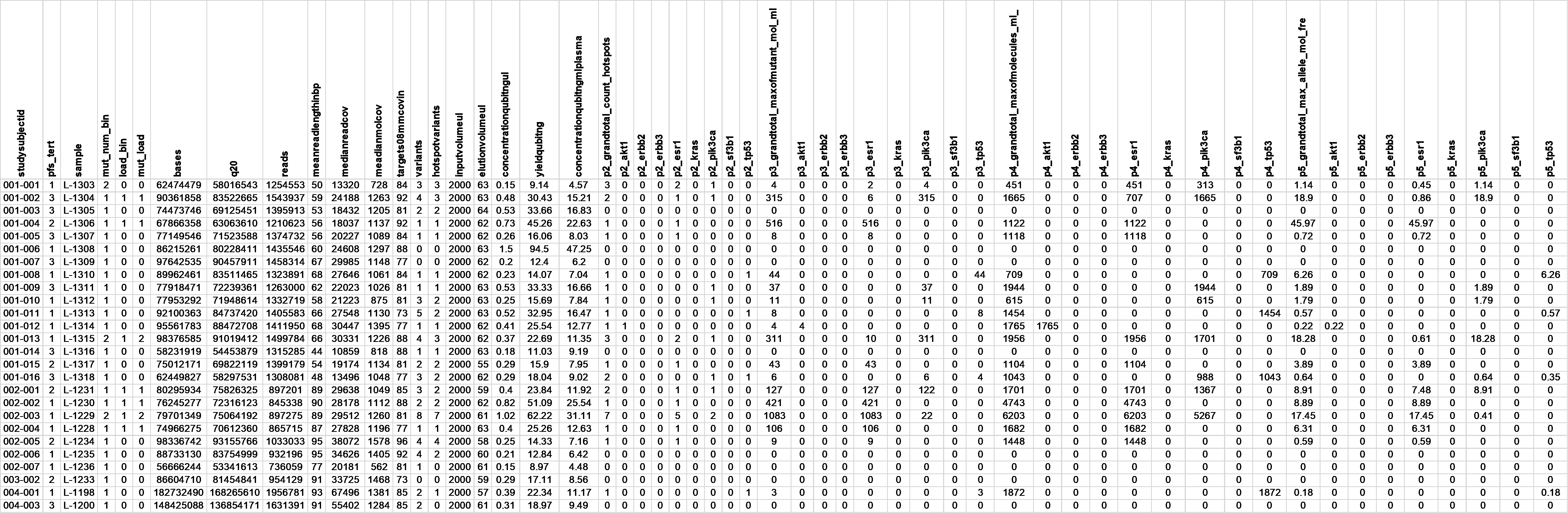

Supplement: Supplementary file 6 — Table S2. Summary and details of NGS results. [file MOL2-14-490-s006.png]

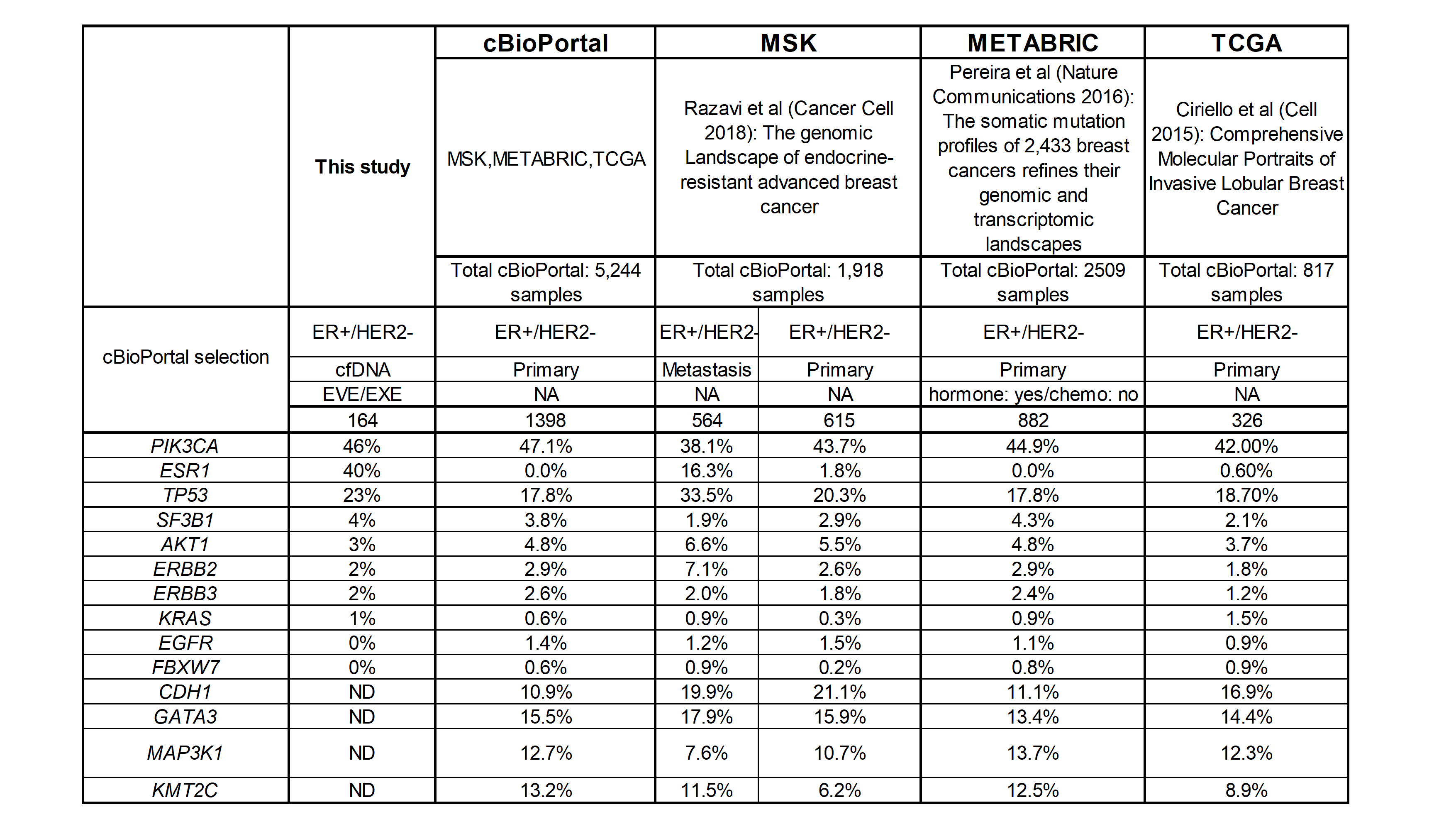

Supplement: Supplementary file 7 — Table S3. In silico database evaluation of Oncomine cfDNA panel genes and most frequently mutated genes of each dataset. [file MOL2-14-490-s007.png]

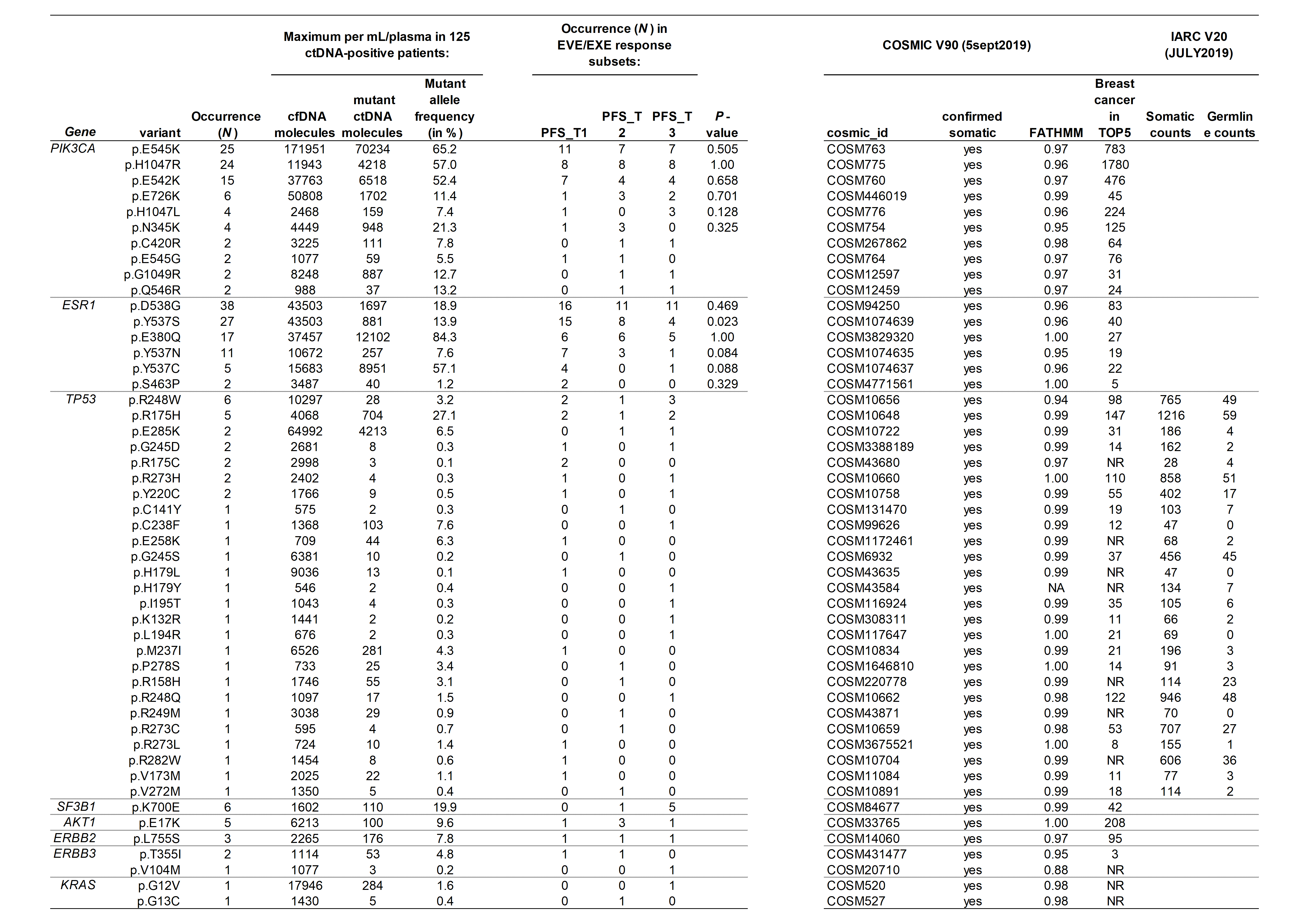

Supplement: Supplementary file 8 — Table S4. List of identified gene hotspot mutations, their occurrence in EVE/EXE response subsets, and their COSMIC and IARC information. [file MOL2-14-490-s008.png]

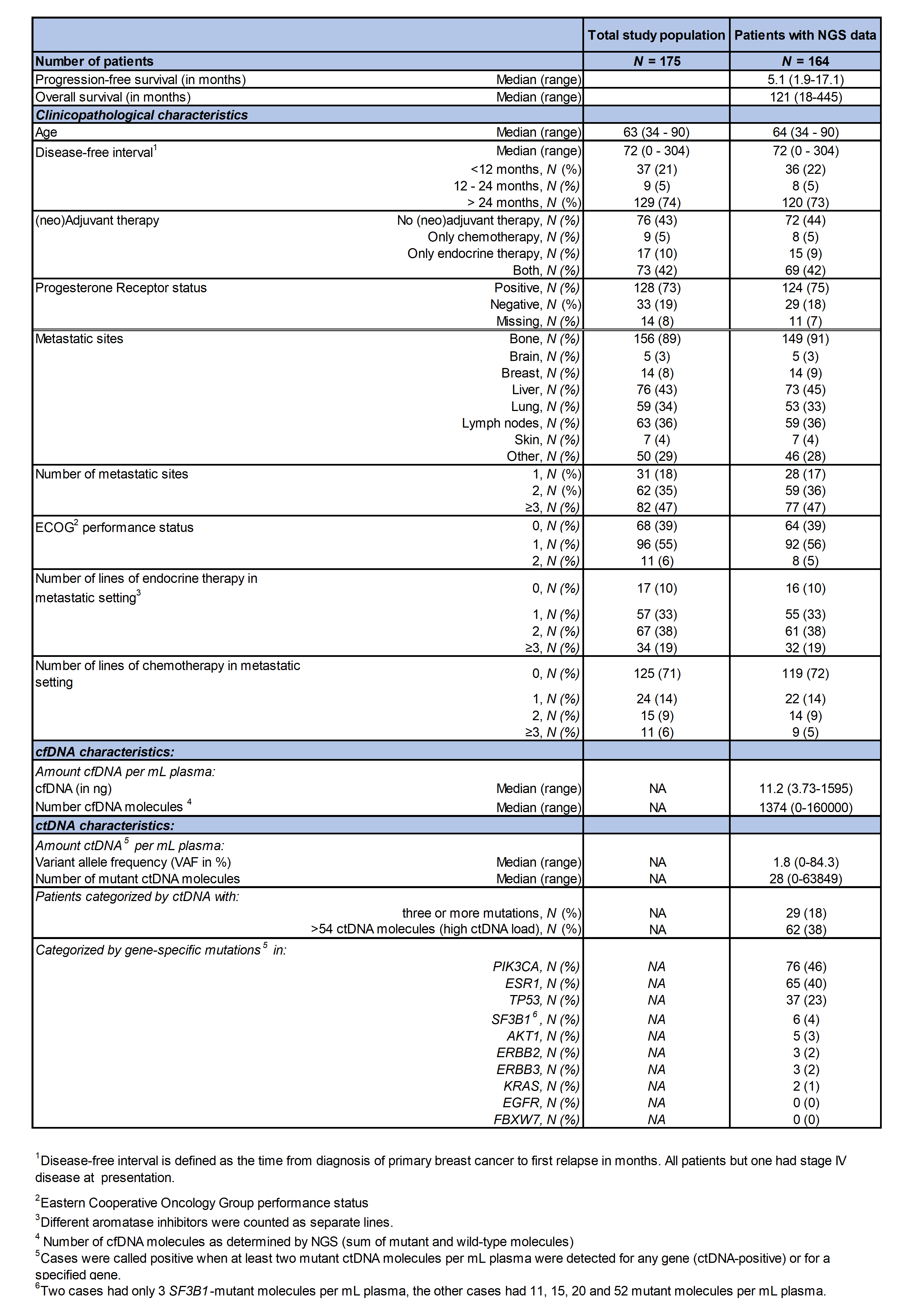

Supplement: Supplementary file 9 — Table S5. Clinical and cfDNA characteristics of total study population and patients with NGS data. [file MOL2-14-490-s009.png]

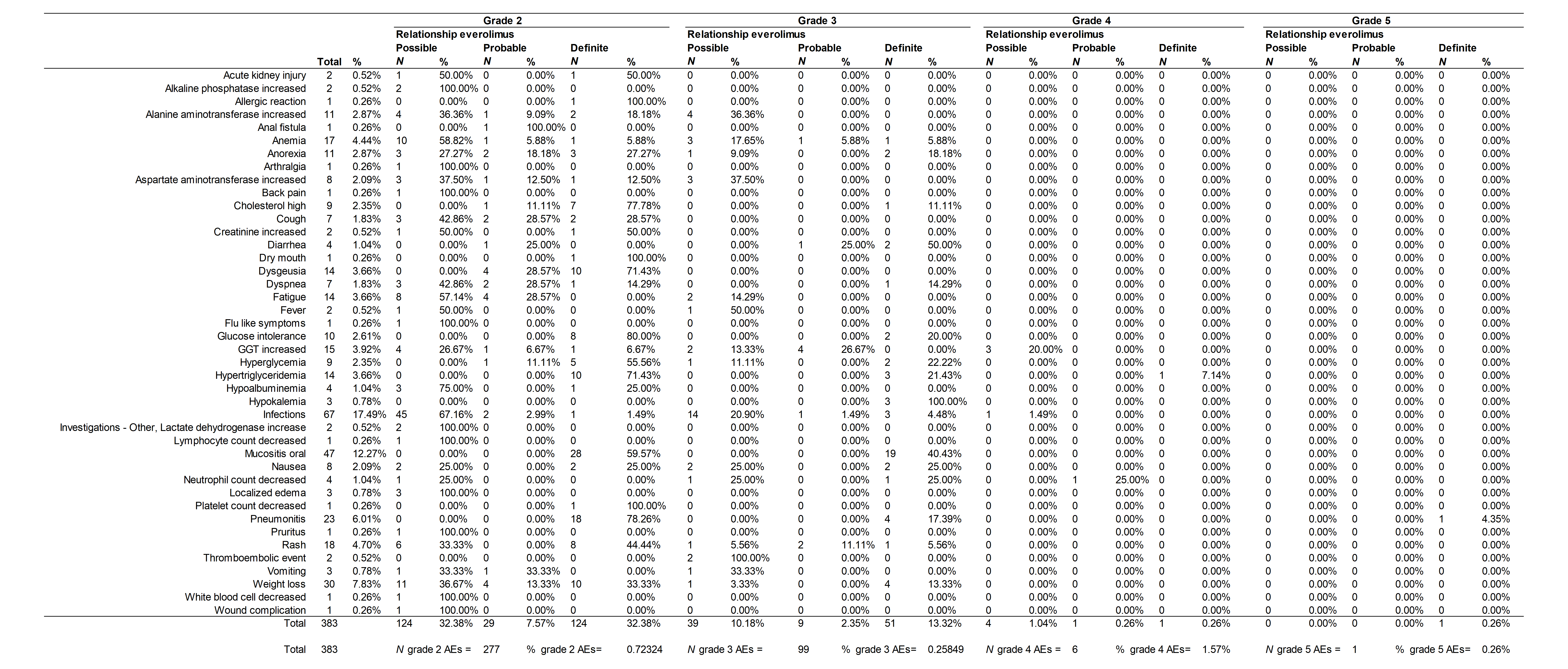

Supplement: Supplementary file 10 — Table S6. Summary of AEs possibly, probably or definitely related to EVE. [file MOL2-14-490-s010.png]

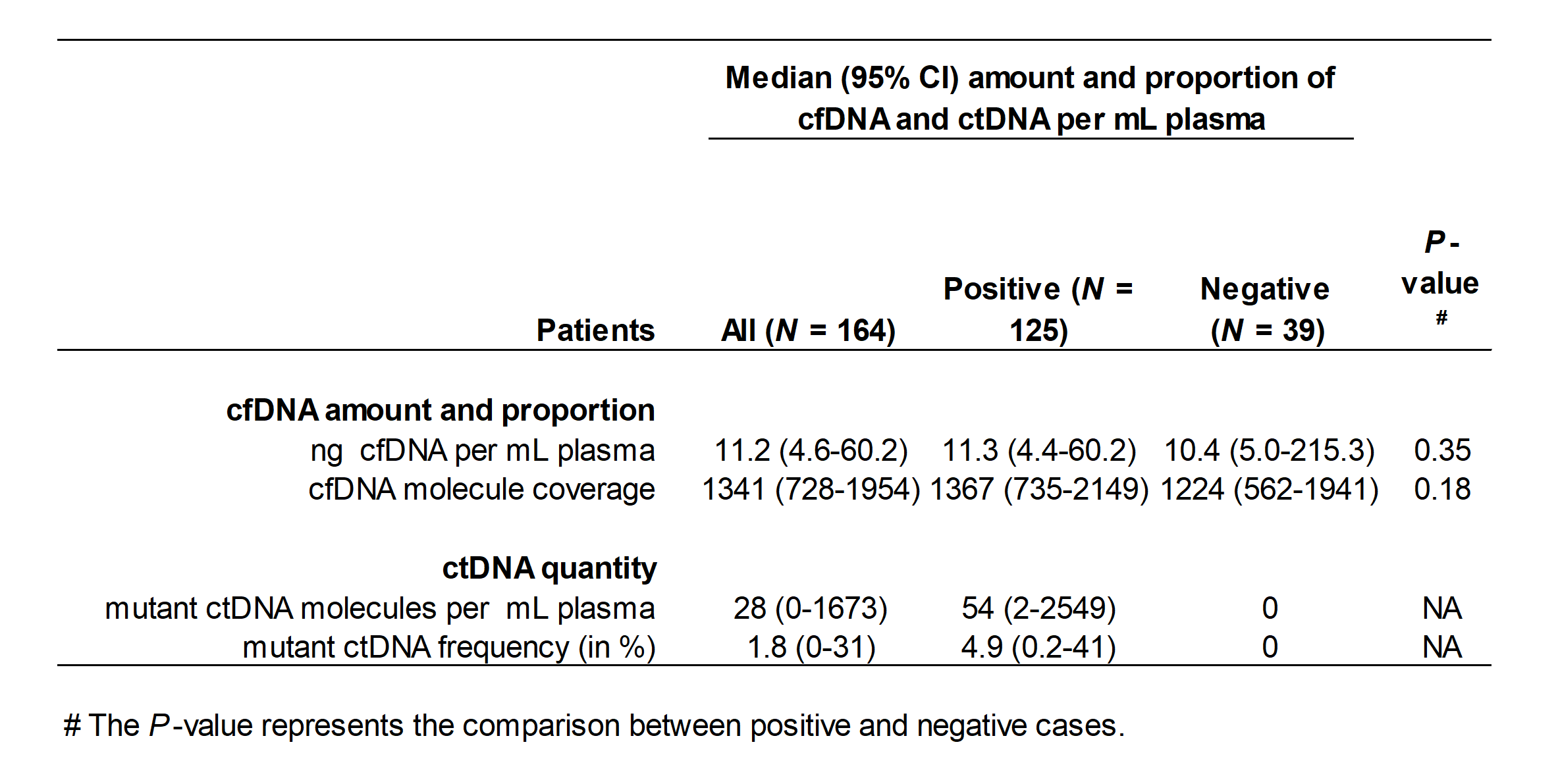

Supplement: Supplementary file 11 — Table S7. Summary of cfDNA and ctDNA characteristics. [file MOL2-14-490-s011.png]

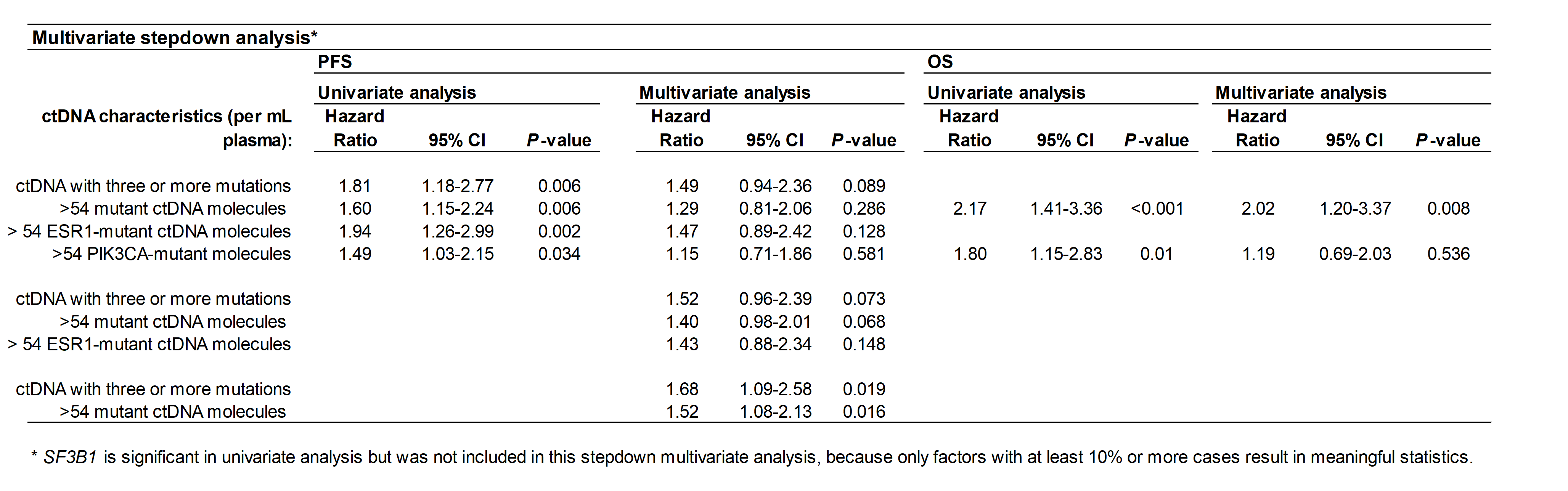

Supplement: Supplementary file 12 — Table S8. Multivariate stepdown analysis. [file MOL2-14-490-s012.png]

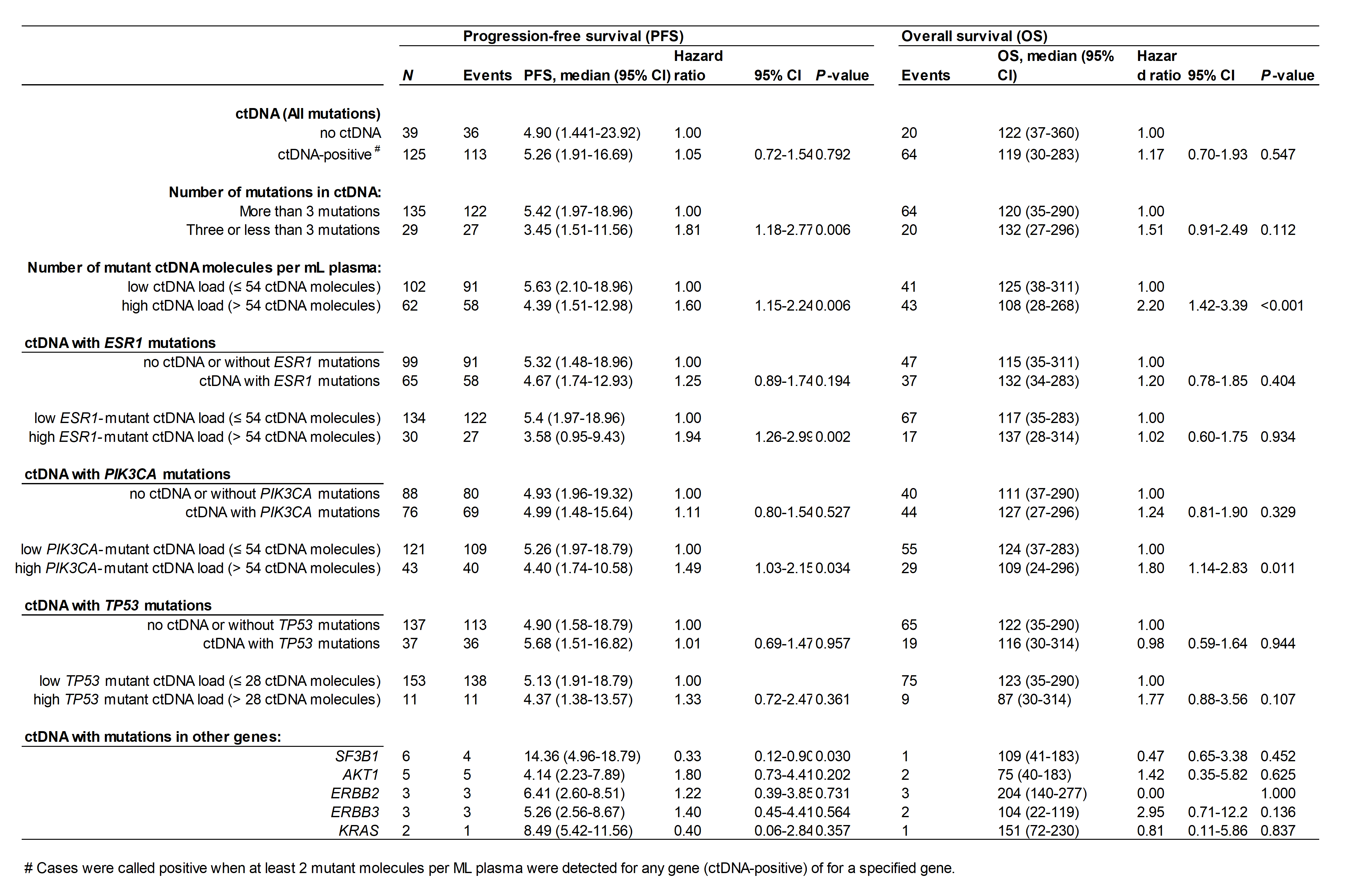

Supplement: Supplementary file 13 — Table S9. Uni‐ & multivariate analysis of ctDNA characteristics for progression‐free and OS. [file MOL2-14-490-s013.png]

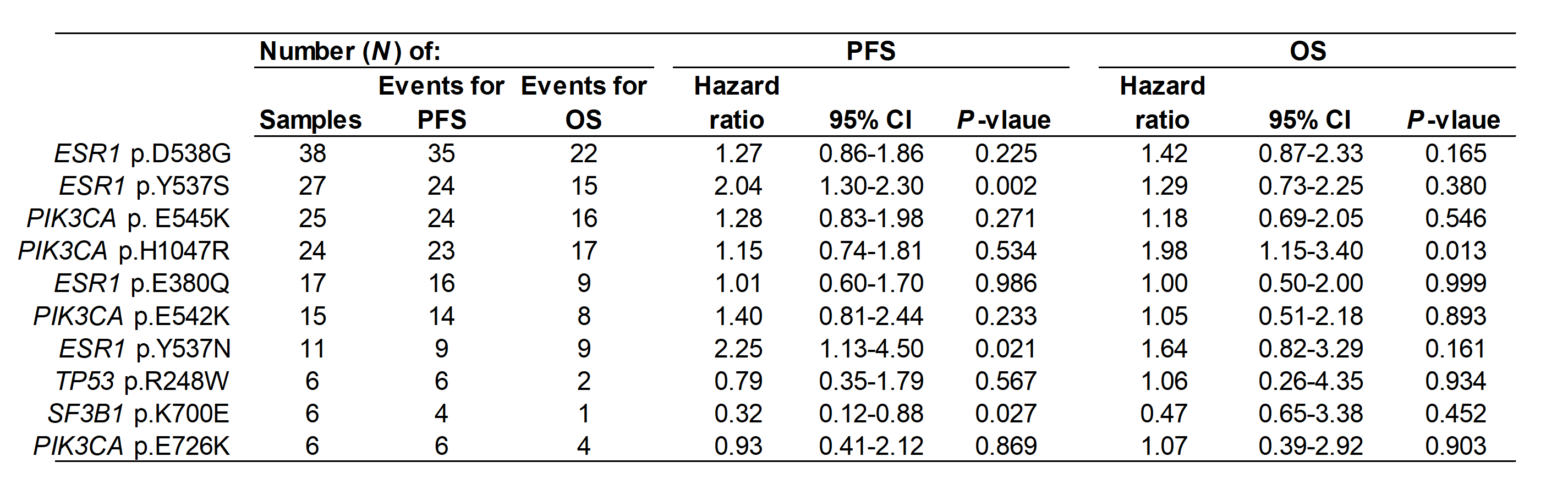

Supplement: Supplementary file 14 — Table S10. Cox regression analyses gene hotspot mutations for progression‐free and OS. [file MOL2-14-490-s014.png]
