# Supplementary material for: High ctDNA molecule numbers relate with poor outcome in advanced ER+, HER2− postmenopausal breast cancer patients treated with everolimus and exemestane
Source: Mol Oncol. 2020 Feb 7;14(3):490–503. doi: 10.1002/1878-0261.12617 (PMC7053245; doi:10.1002/1878-0261.12617)
Supplement: Supplementary file 15 — Appendix S1. Results. [file MOL2-14-490-s015.docx]

**High ctDNA molecule numbers relate with poor outcome in advanced ER+, HER2- postmenopausal breast cancer patients treated with everolimus and exemestane**

Dinja T. Kruger, Maurice P.H.M. Jansen, Inge R.H.M. Konings, Wouter M. Dercksen, Agnes Jager, Jamal Oulad Hadj, Stefan Sleijfer, John W.M. Martens, and Epie Boven

## Supplemental file

Appendix S1**. Results**

***Everolimus dose***

Dose reductions or interruptions of everolimus due to other reasons than progressive disease were frequent since only 40 out of 175 patients were able to complete treatment at the prescribed dose of 10 mg daily. 58 patients started with 5 mg/day due to frailty of whom 34 were able to increase the dose to 10 mg/day according to the protocol. 90% of dose reductions or interruptions were because of toxicity. Other reasons for dose interruptions were due to scheduled surgery or radiotherapy.

### *Characterisation of cfDNA by next generation sequencing*

The median amount of cfDNA in ng and molecular coverage was higher in ctDNA-positive patients (11.3ng; 1367 molecules/mL plasma) compared to ctDNA-negative patients (10.4ng; 1224 molecules/mL plasma) (Figure S3A, Table S5). A single hotspot mutation was observed in 55 patients (Figure S3A), including 25 patients with one *PIK3CA* mutation and 22 patients with one *ESR1* mutation. Two to seven hotspot mutations were detected in the remaining 70 ctDNA-positive patients (Figure S3B), of which 59 patients had mutations in two genes, including 37 patients (23%) with mutations in both *ESR1* and *PIK3CA.* Mutations in *AKT1* and *PIK3CA* were not seen together in ctDNA with two or more mutations (Figure S3B). Figure S3 represents Kaplan-Meier curves showing the relationship between PFS and ctDNA presence (S4A), and presence of *ESR1* (S4B) or *PIK3CA* (S4C) mutations. PFS was prolonged in patients with high numbers of *SF3B1*-mutant ctDNA molecules (*P*=0.048, Figure 1A, Table 1).

### *Pre-treatment plasma ctDNA load and heterogeneity*

Since molecular barcoding was combined with NGS, we were able to quantify the tumour load in blood by the number of mutant molecules (ctDNA molecules) per mL plasma. The tumour load in the 125 ctDNA-positive patients ranged from 2 to 63849 ctDNA molecules per mL plasma (Figure S3A). The mutational spectrum of 10 genes was simultaneously evaluated per pre-treatment cfDNA by our NGS approach, making it possible to explore heterogeneity. In 70 patients two or more mutations were detected in ctDNA and of these, 11 patients had multiple mutations in only one gene. 59 patients had mutations in two or more genes (Figure S3B). For example, one patient had ctDNA with five different *ESR1* mutations and two different *PIK3CA* mutations. In 43 patients, at least a two-fold difference in amount of ctDNA molecules between two genes was observed. For instance, two cases had more than 1000 ctDNA molecules with an *ESR1* mutation but only around 100 ctDNA molecules with a *PIK3CA* mutation, and one of these also had ~10 ctDNA molecules with a *TP53* mutation. When focusing on the 37 patients with *ESR1* and *PIK3CA* mutations, a two-fold or greater difference in the number of ctDNA molecules per mL plasma between these genes was observed in 28 patients. Of these, in 25 patients the *PIK3CA* mutation was the major clone (Figure S3B and S3C).

**Legends Supplemental Figures and Tables**

**Figure S1 – Biomarker workflow: Plasma cfDNA isolation and ctDNA characterization by NGS and molecular barcoding**.

**Figure S2 – Study design: Setting and participants of the Everolimus plus Exemestane Biomarker study**.

**Figure S3 – Circulating tumour DNA (ctDNA) characteristics: Number of mutations and ctDNA load.** Missense hotspot mutations were detected in eight of 10 genes in two or more ctDNA molecules per mL plasma for 125/164 patients (Figure S3A). The amount of cfDNA in ng per ml plasma per patient is represented by the dotted line. When the number of mutations per patient was analysed, 55 patients had only one missense hotspot mutation, whereas two or more mutations were detected in 70 patients (red bars Figure S3A). The ctDNA molecule numbers per mL plasma are shown by the grey bars in Figure S3A. For patients with two or more mutations, the dominant mutation is presented (Figure S3B). This figure represents mutated genes specified by colour: ESR1 (yellow), PIK3CA (green), TP53 (brown), SF3B1 (dark blue), AKT1 (blue), ERBB2 (orange), ERBB3 (white), and KRAS (light blue). Mutations in ESR1 and PIK3CA were most frequently observed. 22 patients had multiple ESR1 mutations and nine patients multiple PIK3CA mutations (Figure S3B).

**Figure S4 – Kaplan-Meier survival curves evaluation for ctDNA and its relationship with progression-free survival (PFS) on EVE/EXE.** Kaplan-Meier survival curves evaluation for ctDNA and its relationship with progression-free survival (PFS) on EVE/EXE. The relationships were examined for lack or presence of ctDNA (at least 2 ctDNA molecules per mL plasma) (A) and for ctDNA with ESR1 or PIK3CA mutations (Figures B & C). No/low ctDNA-load is defined by ≤ 54 mutant ctDNA molecules per mL plasma, high ctDNA-load has >54 mutant ctDNA molecules per mL plasma. Same definition is used for low/high ctDNA-load with ESR1 or PIK3CA mutations. The plots presents next to patients at risk also P-values based on logrank test.

**Table S1 – List of participating hospitals**

**Table S2 – Summary and details of NGS results**

**Table S3 – *In silico* database evaluation of Oncomine cfDNA panel genes and most frequently mutated genes of each dataset.**

**Table S4 – List of identified gene hotspot mutations, their occurrence in EVE/EXE response subsets, and their COSMIC and IARC information**

**Table S5 – Clinical and cfDNA characteristics of total study population and patients with NGS data.**

**Table S6 – Summary of adverse events possibly, probably or definitely related to everolimus.**

**Table S7 - Summary of cfDNA and ctDNA characteristics**

**Table S8 – Multivariate stepdown analysis**

**Table S9 – Uni- & multivariate analysis of ctDNA characteristics for progression-free and overall survival**

**Table S10 – Cox regression analyses gene hotspot mutations for progression-free and overall survival**
